# Glass Polymorphism in Hyperquenched Aqueous LiCl Solutions

**DOI:** 10.1021/acs.jpcb.3c01030

**Published:** 2023-04-07

**Authors:** Johannes Giebelmann, Johannes Bachler, Thomas Loerting

**Affiliations:** Institute of Physical Chemistry, University of Innsbruck, Innrain 52c, A-6020 Innsbruck, Austria

## Abstract

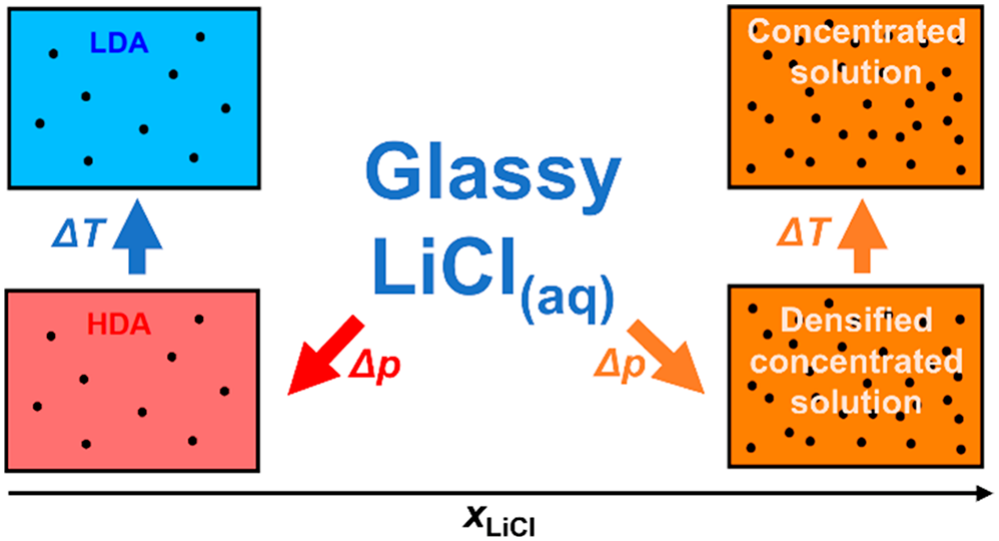

We investigate the glass polymorphism of dilute LiCl–H_2_O in the composition range of 0–5.8 mol % LiCl. The solutions are vitrified at ambient pressure (requires hyperquenching with ∼10^6^ K s^–1^) and transformed to their high-density state using a special high-pressure annealing protocol. Ex situ characterization was performed via isobaric heating experiments using X-ray diffraction and differential scanning calorimetry. We observe signatures from a distinct high-density and a distinct low-density glass for all solutions with a mole fraction *x*_LiCl_ of ≤ 4.3 mol %, where the most notable are (i) the jumplike polyamorphic transition from high-density to low-density glass and (ii) two well-separated glass-to-liquid transitions *T*_g,1_ and *T*_g,2_, each pertaining to one glass polymorph. These features are absent for solutions with *x*_LiCl_ ≥ 5.8 mol %, which show only continuous densification and relaxation behavior. That is, a switch from water-dominated to solute-dominated region occurs between 4.3 mol % LiCl and 5.8 mol % LiCl. For the water-dominated region, we find that LiCl has a huge impact only on the low-density form. This is manifested as a shift in halo peak position to denser local structures, a lowering of *T*_g,1_, and a significant change in relaxation dynamics. These effects of LiCl are observed both for hyperquenched samples and low-density samples obtained via heating of the high-density glasses, suggesting path independence. Such behavior further necessitates that LiCl is distributed homogeneously in the low-density glass. This contrasts earlier studies in which structural heterogeneity is claimed: ions were believed to be surrounded by only high-density states, thereby enforcing a phase separation into ion-rich high-density and ion-poor low-density glasses. We speculate the difference arises from the difference in cooling rates, which are higher by at least 1 order of magnitude in our case.

## 1 Introduction

Amorphous ices play important roles in Nature. They are the most abundant form of water in the interstellar space and are vital to a lot of astrochemical processes, possibly including the formation of organic molecules such as amino acids.^1^ But there are also applications that make use of amorphous ices, such as cryo-electron microscopy.^2^ Three different forms of amorphous ice are known, namely low-density amorphous ice (LDA),^3^ high-density amorphous ice (HDA),^4,5^ and very-high-density amorphous ice (VHDA).^6^ This phenomenon is termed *polyamorphism*. Especially the relation between LDA and HDA has sparked great interest in the field, because they can be interconverted into another reversibly via an apparent first-order transition.^3^ According to the prominent liquid–liquid critical point (LLCP) scenario, LDA and HDA are predicted to be the glassy states thermodynamically linked to two distinct liquids: low-density liquid (LDL) and high-density liquid (HDL), respectively.^7^ Consequently, the two polyamorphs exhibit different calorimetric glass-transition temperatures at ambient pressure, where the glass-transition temperature of LDA (*T*_g,1_) is located at 136 K^8−10^ and the glass transition of HDA (*T*_g,2_) is located at 116 K.^11^ However, the scenario was contested for a long time, and only in recent years, much more evidence in favor of the LLCP model has been gathered.^12^ This includes strong experimental proof that LDA and HDA are truly glassy states, which turn into ultraviscous liquids upon heating.^13,14^

Another important aspect in the field is how their properties are affected by solutes. There is a vast number of studies on different solutes and their effects on water polyamorphism. These effects include, but are not limited to, shifts in glass-transition temperatures,^15−18^ shifts in onset temperatures and onset pressures of polyamorphic transitions,^19−22^ vanishing of polyamorphism,^23,24^ suppression of crystallization,^15,25^ and phase separation.^23,26,31^ Naturally, the type and strength of these effects depend on the kind of solute added. For detailed information, please see our recent review on the topic.^25^

In this regard, the arguably most thoroughly investigated system is LiCl–H_2_O. First and foremost, LiCl facilitates glass formation by impeding the crystallization of ice I.^15,26^ Thus, crystallization can be easily avoided when adding a sufficient fraction of salt. Tanaka and Kobayashi^27^ showed that, at certain mole fractions (12.5–25 mol % LiCl or *R* = 7–3, where *R* is defined as moles water per moles of solute), glasses are obtained even by cooling with rates as low as 0.1 K min^–1^. However, for lower mole fractions, the critical cooling rate increases very rapidly. This was demonstrated by Angell and Sare,^15^ who determined the glass-forming region by quenching solutions at a rate of ∼1000 K min^–1^. They found that solutions with <9 mol % of LiCl (*R* = 10) can no longer be vitrified with easily experimental accessible cooling rates. Interestingly, there is no sign of polyamorphism for these concentrated glasses.^28^ Instead, the solutions end up in a state structurally resembling HDA^29,30^ that exhibits a single glass-to-liquid transition temperature (*T*_*g*__,conc_). This contrasts the case of pure glassy water, which can be encountered in either a low-density state (*T*_*g*__,1_) or a high-density state (*T*_g,2_).

Only at even lower concentrations, the polyamorphic behavior of water is restored, and the influence of solutes on LDA and HDA can be studied. It is now necessary to distinguish between the vitrification procedures that produce low-density glasses and the ones that yield high-density glasses. The former are usually achieved by ultrafast cooling (∼10^6^ K s^–1^) of the solution at ambient or subambient pressure (“hyperquenching”), whereas the latter require pressurization and fast cooling (“pressure vitrification”).

Hofer et al.^17^ used the hyperquenching technique, which makes vitrification even possible for pure water. The material obtained by hyperquenching pure water is termed *hyperquenched glassy water* (HGW) or *hyperquenched glassy solution* (HGS) in the case of the LiCl solutions. The authors revealed a complex concentration dependency of the calorimetric glass-transition temperature *T*_*g*__,1_ comprising a minimum at ∼3 mol % (*R* ≈ 32), followed by a steep increase at ∼4 and 6 mol % (*R* = 24–16) back to ∼136 K. This result was speculated to be due to plasticization and antiplasticization of the hydrogen-bond network.

On the other hand, Kanno used pressure-vitrification (PVI) at 0.25 GPa and cooling rates of 180 K min^–1^, which allows for vitrification down to ∼5 mol % (*R* = 19).^31^ That is, in contrast to hyperquenching, highly diluted solutions could not be accessed. In his study, two exothermic events related to the polyamorphic transition from LiCl-HDA to LiCl-LDA were recorded upon heating at ambient pressure. The *T*_g,2_ of LiCl-HDA was not detected, since it can only be observed after using appropriate high-pressure relaxation protocols unknown at that time.^11,20^ Yet, upon heating the high-density glass at elevated pressures, Kanno^31^ identified two consecutive but distinct glass-transition temperatures. Because of the lack of a polyamorphic transition separating the two, they are assigned to two immiscible liquids: one of low LiCl concentration and one of high LiCl concentration. However, this assignment necessitates a preceding phase separation of the solution to solute-rich and solute-poor regions, which should occur already during the cooling process. The possibility of such a phase separation and the underlying processes have been discussed vividly ever since.

In particular, Suzuki and Mishima^23,32^ advocate this view: They scrutinized the ambient pressure behavior of emulsified pressure-vitrified solutions with molar fractions between 2 and 10 mol % (*R* = 49–9). Upon heating the dilute glassy solution, they observe the polyamorphic transition of LiCl-HDA similarly to that reported by Kanno.^31^ Yet, the vibrational structure of the resulting product can be expressed as a linear combination of pure LDA and a concentrated solution (CS) of LiCl–H_2_O. This indicates a phase separation either during pressure vitrification or triggered by the polyamorphic transition. Suzuki and Mishima find hints of phase separation in both LiCl-LDA^33^ and LiCl-HDA,^34^ although they are more pronounced for the low-density glass. Therefore, they propose that LiCl is immiscible with LDA, and LiCl-HDA must experience a polyamorphic phase separation into pure LDA and concentrated solution. This notion received support from MD simulations conducted on a coarse-grained model of water (mW), in which water is represented as a single particle that prefers tetrahedral binding.^35^ In solutions of LiCl in mW, a so-called nanophase segregation upon cooling of the solution was encountered.^36^ It is stated that this behavior is linked to water’s polyamorphism since LDL forms as the temperature decreases and the ions that do not fit into the tetrahedral network are expelled, thereby forming a concentrated solution.

However, it is not clear how this hypothesis is compatible with the results of Hofer et al.^17^ and Kanno.^31^ The former find a significant lowering in the glass-transition temperature, speaking in favor of homogeneous vitrification where LiCl ions affect *T*_g,1_ of the hyperquenched glassy matrix. The latter finds two glass transitions even under high-pressure conditions, implying that also LiCl-HDA represents an inhomogeneous high-density state, which presumably remains phase-separated after the polyamorphic transition. These apparent contradictions could be due to the fact that, for glasses, the exact thermal history (i.e., the preparation route) has a large influence on their properties.

In the present study, we explore a novel pathway of preparing high-density glasses: the pressurization of hyperquenched aqueous LiCl solution, a method that we developed for pure water in a recent study.^14^ This method allows studying LiCl-HDA even at the lowest molar fractions and without the need of an emulsifying agent. Specifically, we hyperquenched solutions between 0.5 and 5.8 mol % (*R* = 199–16.2) of LiCl and compressed them isothermally to yield high-density glasses. Dilatometric curves were recorded during compression. After high-pressure annealing,^14^ the samples were quenched and recovered at ambient pressure, at which ex situ X-ray diffraction (XRD) and differential scanning calorimetry (DSC) measurements were performed. In the following sections, we thoroughly discuss the phase behavior of the polyamorphs, including their glass-to-liquid transitions. We attempt to interpret our results on the basis of both homogeneous vitrification upon hyperquenching and a possible liquid–liquid immiscibility leading to a phase-separated glass. The nature and mechanism of the polyamorphic transition are discussed in a companion study.^37^

## 2 Experimental Methods

Aqueous LiCl solutions were prepared by adding anhydrous LiCl to Milli-Q water (Millipore). Complete vitrification of solutions was realized through the hyperquenching method using the same optimized setup as that described in the work by Kohl et al.^38^ Briefly, an aerosol of each solution was formed through nebulization, employing an ultrasonic nebulizer operating at 3 MHz (LKB Instruments, Model 108). It produces droplets with a mean diameter of ∼3 μm. Using dry nitrogen as a carrier gas, these droplets were then transported through an aerosol hose that was cooled using an ice bath. This measure removes larger droplets (after coalescence) and lowers the water vapor pressure, thereby eliminating the accompanying vapor deposition. After the ice bath, the droplets are carried to a high-vacuum chamber, which they enter through a 300 μm orifice. The vacuum is achieved by the combination of a powerful cryo pump (Air Products, Model DE-208L) and a turbo-molecular pump (Leybold-Heraeus, Model Turbovac 360). In order to avoid possible oil contaminations, a roots pump (Pfeiffer Vacuum, Model ACP15) is preferred over a rotary vane pump as a prepump. Because of the large pressure difference, the aerosol droplets hit a liquid-nitrogen-cooled cryo-plate made from oxygen-free high-conductivity copper with ultrasonic speeds. This results in cooling rates of >10^6^ K s^–1^, which is sufficient to yield more than ∼95% vitrified pure water and 100% vitrified solutions. After ∼30 min of deposition time, a deposit 1–2 mm thick was recovered from the apparatus and stored under liquid nitrogen. To ensure that the obtained deposits were amorphous, they were checked using DSC and XRD.

The hyperquenched solutions (0–5.8 mol %; *R* = ∞–16.2) and a CS (12.2 mol %, *R* = 7.2, quenched in a container cooled with liquid nitrogen), were densified as described in a previous publication.^14^ For each batch, ∼200−400 mg of hyperquenched solution was removed from the cryo-plate and placed into an indium container fitted to the 8 mm bore of the piston–cylinder setup. The compression cell remained immersed in liquid nitrogen during the transfer process. For compression, an uniaxial force was applied using a material testing machine (Zwick Roell, Model BZ100/TL3S). The temperature during compression was controlled through an adjustable liquid nitrogen cooling system in combination with two heating rods along with a Pt-100 sensor that were inserted in the cell. Initially, each sample was pressurized to 1.9 GPa at 77 K with a compression rate of ∼40 MPa min^–1^. Then, it was warmed to 175 K at 1.9 GPa with a heating rate of 5 K min^–1^, immediately followed by cooling back to 140 K as quickly as possible. Afterward, each sample was decompressed at 140 K to 0.15 GPa employing a decompression rate of ∼20 MPa min^–1^. At this pressure, the sample is quenched to 77 K and subsequently recovered to ambient pressure. During this recovery procedure, the sample becomes kinetically arrested in the high-pressure state and remains there, even after the pressure is released.^39^ This allows ex situ characterization with methods that are limited to ambient pressure.

Ex situ powder XRD was carried out using a D8 Bruker Advance X-ray diffractometer equipped with a low-temperature chamber (FMB Oxford, Ltd.) that allows measurements under cryogenic conditions. The temperature was controlled with a two-stage helium cryostat, combined with a silicon diode and resistive heating elements. This setup, which we also used in previous publications,^13,14^ allows for precise temperature control between 20 K and 300 K. The incident wavelength was λ = 0.154178 nm (Cu Kα), and a Goebel mirror was used, along with a LynxEye XE-T array detector. The samples were placed onto custom-made copper sample holders that either allow for directly loading a cryo-plate of hyperquenched glass or a pellet of recovered compressed sample. The 12.2 mol % slow-quenched CS was loaded as a fine powder on a different copper sample holder. The sample transfer was conducted at liquid nitrogen temperature and with minimal exposure to air.

Ex situ DSC measurements were conducted using a PerkinElmer Model DSC8000 system that was calibrated with indium, adamantane, and cyclopentane for heating and cooling rates of 10 and 30 K min^–1^. Transition temperatures can be reproduced with an accuracy of ±1 K. Under liquid nitrogen, about 10 mg of sample were loaded into an aluminum crucible. The crucible was sealed and loaded into the instrument precooled to 93 K. The following protocol was performed for five different heating rates ranging from 10 K min^–1^ to 50 K min^–1^ and a constant cooling rate of 30 K min^–1^ each. First, each sample was heated to 143–153 K (depending on the heating rate) to fully transform the high-density sample to its low-density state. Then, an annealing step was performed at 128 K for 30 min after which the sample was recooled to 93 K. The additional annealing step is mandatory for unveiling the glass transitions of hyperquenched glassy water and solutions.^8^ Subsequently, the sample was heated to room temperature to observe cold crystallization of the low-density state and melting. For reference and baseline determination, each sample was cooled to 93 K and heated to room temperature again. The sample mass has been determined by comparing the enthalpy of fusion in the second heating scan with the corresponding value calculated from the data of Monnin et al.^40^

## 3 Results

### 3.1 Dilatometry

**Figure 1:**
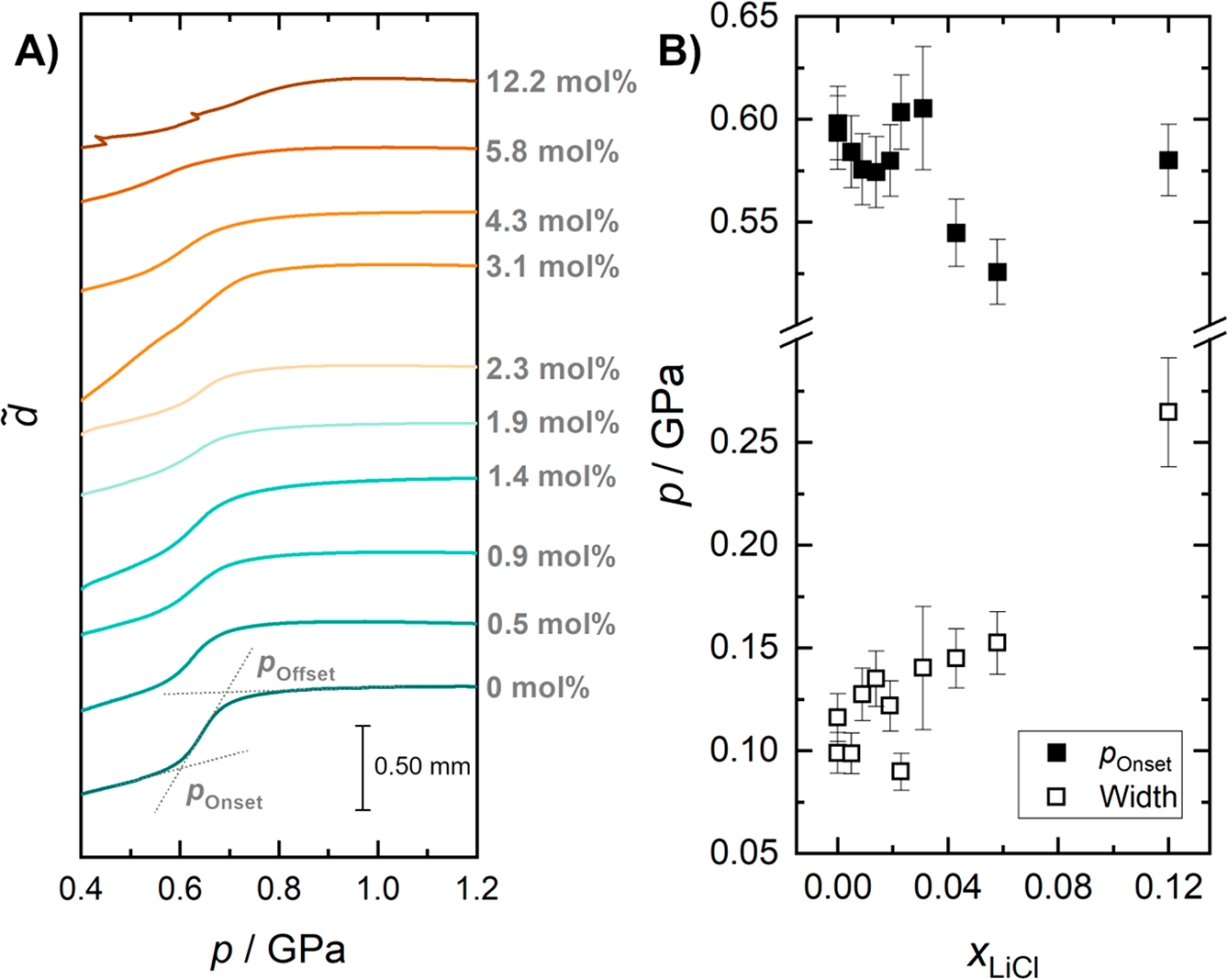
(A) Piston displacement *d̃* vs pressure of the hyperquenched samples (0–5.8 mol %, *R* = ∞–16.2) and the CS (12.2 mol %, *R* = 7.2) at 77 K. The curves have been corrected for the slope at ∼1 GPa and are stacked for clarity. Roughly 200–400 mg were loaded into the cell, but the exact sample mass is unknown. Therefore, no normalization relative to the amount of sample could be performed, and absolute densification cannot be quantified. A straight line at ∼1 GPa, after the steplike change in density, was subtracted from the raw data (shown in Figure S1 in the Supporting Information) to compensate for the compressibility of the high-density glasses. For pure water, a change in piston displacement of ∼0.50 mm was observed for the steplike feature, which implies that ∼390 mg of sample were loaded (see Figure S1). The onset/offset pressure *p*_Onset_/*p*_Offset_ is defined by the intersection of the extrapolated baseline with a tangent aligned to the inflection point of the sigmoidal increase as indicated in the curve for pure water. (B) Onset pressure (filled squares) and width (empty squares) of the polyamorphic transition with increasing mole fraction of LiCl, where width is defined as *p*_Offset_ – *p*_Onset_.

Figure 1A shows the piston displacement *d̃* of the hyperquenched solutions (0–5.8 mol %, *R* = ∞–16.2) and a reference CS (12.2 mol %, *R* = 7.2) at 77 K, as a function of pressure. For pure HGW, a steplike feature is observed at an onset pressure *p*_Onset_ of 0.60 ± 0.02 GPa. This sudden densification is a hallmark of the polyamorphic LDA → HDA transition of hyperquenched glassy water (HGW) to densified hyperquenched glassy water (dHGW) that is usually observed between 0.6 and 0.7 GPa.^13^ Please note that, based on our recent findings,^13,14^ we will no longer differentiate between HGW and LDA, as well as d-HGW and HDA but use the terms interchangeably. The slope at 1.0 GPa, which relates to the isothermal compressibility of HDA, has been zeroed in Figure 1A by subtracting a straight line. At *p* < 0.6 GPa, the curve slopes upward, which implies that LDA is more compressible than HDA. This is a consequence of the more-open structure of LDA, in which no interstitial sites between first and second hydration shell are occupied.

For hyperquenched LiCl solutions up to 2.3 mol % (*R* = 42.5), *p*_Onset_ and the width of the transition, defined as *p*_Offset_ – *p*_Onset_ (see Figure 1B), are barely affected when comparing to pure water. For the 3.1 mol % (*R* = 31.3) sample, the identification of *p*_Onset_ is difficult because of the steep slope of *d̃* between 0.4 and 0.6 GPa. We suspect that this is due to some trapped air inside the sample that is slowly squished out. Other than for the 3.1 mol % sample, the compressibility at <0.6 GPa is hardly affected by the addition of LiCl. That is, the compressibility of LiCl-HGW and LiCl-dHGW are still quite similar to the compressibility of pure HGW and pure dHGW. At 4.3 mol % (*R* = 22.3) and 5.8 mol % (*R* = 16.2), the step-like feature can be distinguished again but is shifted to slightly lower *p*_Onset_ values when compared to lower mole fractions. In addition, the transition broadens and flattens when compared to solutions with mole fractions of <2.3 mol %. For the slowly cooled CS of 12.2 mol %, the step-like transition is replaced by a very broad and continuous transition at slightly higher pressures.

From these results, we infer a polyamorphic transition of the hyperquenched LiCl-solutions, LiCl-HGW (or LiCl-LDA), to the high-density glass, LiCl-dHGW (or LiCl-HDA) upon compression at 77 K for all hyperquenched samples (0–5.8 mol %, *R* = ∞–16.2). In contrast, CS (12.2 mol %, *R* = 7.2) densifies continuously with no sign of genuine polyamorphism, in agreement with the results presented by Suzuki and Mishima.^28^ In addition, we emphasize that we do not see any signs of crystalline ice in the hyperquenched sample. Ice I would experience pressure-induced amorphization (PIA) near 1.0 GPa both for pure water and for solutions with molar fractions of <10 mol % (*R* > 9).^19,20^ No step-like densification arising from PIA is seen in Figure 1A near 1.0 GPa, which means our samples do not contain significant amounts of ice I_h_.

However, from observation of densification alone, which is closely related to observing molar sample volume, we cannot deduce whether the polyamorphic transition starts from the phase-separated or from the homogeneous glass (LiCl-HGW). This issue is tackled based on XRD and calorimetry experiments.

### 3.2 X-ray Diffraction

**Figure 2:**
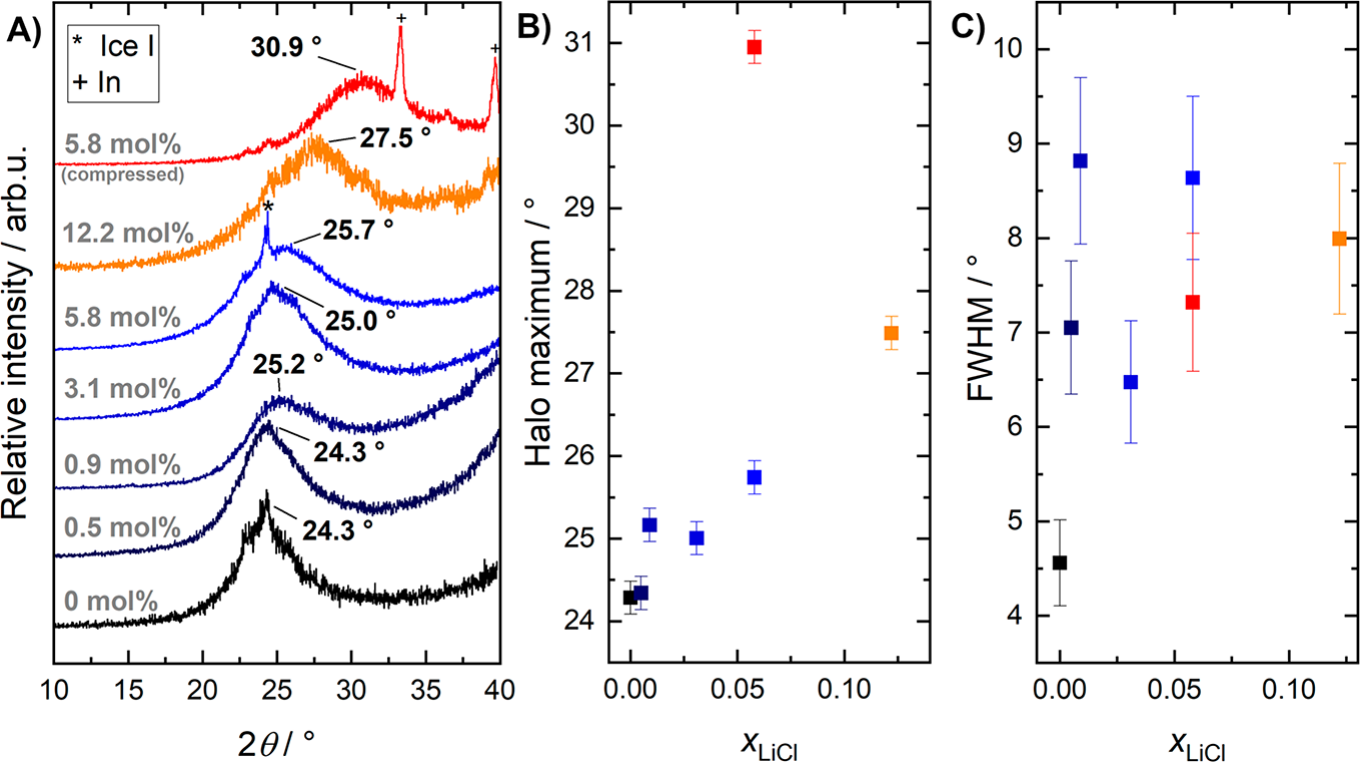
(A) X-ray diffractograms of hyperquenched LiCl solutions before (different shades of blue) and after (red) compression. For comparison, the diffractogram of the slowly quenched CS (orange) is shown as well. All samples were measured as pellets at 80 K, except the CS which was loaded as a powder (see the Methods section). The diffractograms are stacked for clarity and normalized relative to the halo peak height at the labeled peak maximum. Bragg reflexes that correspond to impurities of ice I or indium were marked accordingly. (B) Position of the halo peak maximum as a function of the mole fraction of LiCl (*x*_LiCl_). (C) Full width at half-maximum (fwhm) of the halo peaks shown in panel (A).

X-ray diffractograms of hyperquenched solutions, CS, and a densified hyperquenched solution are shown in Figure 2A. All of them are dominated by a broad halo peak, where some of them contain weak Bragg peaks that stem from indium or ice I. The former is observed in diffractograms of compressed samples because such samples are encapsulated in indium, where the entire sample, including indium, was loaded into the instrument. Traces of ice I in hyperquenched samples are present because of either condensation of water vapor from ambient air or inadvertent heating (and, consequently, crystallization) of the uppermost layers of glass during the sample transfer process. The former leads to ice I_h_, and the latter leads to stacking disordered ice I_sd_, which is mostly cubic ice with some hexagonal stacking faults. The weak intensity of the Bragg peaks associated with ice I does not allow us to determine with confidence whether traces of I_h_ or I_sd_ are present on the samples.

The broad halo peaks, centered at 2θ ≈ 24°–31° (Cu K_α,1_), are characteristic of amorphous ices^4,41^ and indicate that also the hyperquenched solutions are amorphous. For pure HGW, the maximum of the halo peak (Figure 2B) is found at 24.3° ± 0.2°, which fits the literature value well and reflects that HGW is an LDA-type glass.^43^ For LiCl-HGW, small shifts in the halo-peak maximum to higher angles are observed. At 5.8 mol % (*R* = 16.2), the maximum shifts by roughly 1.4° to 25.7° ± 0.2° toward the position in HDA (30.3° ± 0.2°).^13^ For CS (12.2 mol %, *R* = 7.2), the shift is even larger and amounts to a shift of 3.2° to 27.5° ± 0.2°. Interestingly, the full width at half-maximum (fwhm) increases substantially once LiCl is added: It is roughly 4.6° for pure HGW, then jumps up to 7.0° at 0.5 mol % (*R* = 110) and reaches ∼8.6° at 5.8 mol % (*R* = 16.2; see Figure 2C). Such an increase indicates that the distribution of local structures becomes broader when LiCl is added. More different O–O distances are present in LiCl-HGW than in the low-entropy, tetrahedral LDA. This is consistent with radial distribution functions (RDF) determined via neutron diffraction on LiCl-HGW (2.4 mol %, *R* = 40.7).^44^ Thus, we infer that while hyperquenched LiCl solutions are structurally similar to LDA and can be considered low-density glasses up to 5.8 mol %, they appear to become slightly more HDA-like. Further increasing the mole fraction causes the glass to become even more HDA-like, as observed in the case of the CS. This gradual shift toward an HDA state implies that at least some LiCl can be integrated into the LDA matrix. If the solutions had phase-separated upon hyperquenching, we would have expected the coexistence of pure LDA and CS (HDA-like). In terms of the XRD patterns in Figure 2A, we would expect two distinct halo peaks (one pertaining to LDA and the other to HDA), as was observed by Winkel et al. for a sample composed of both LDA and HDA.^45^

**Figure 3:**
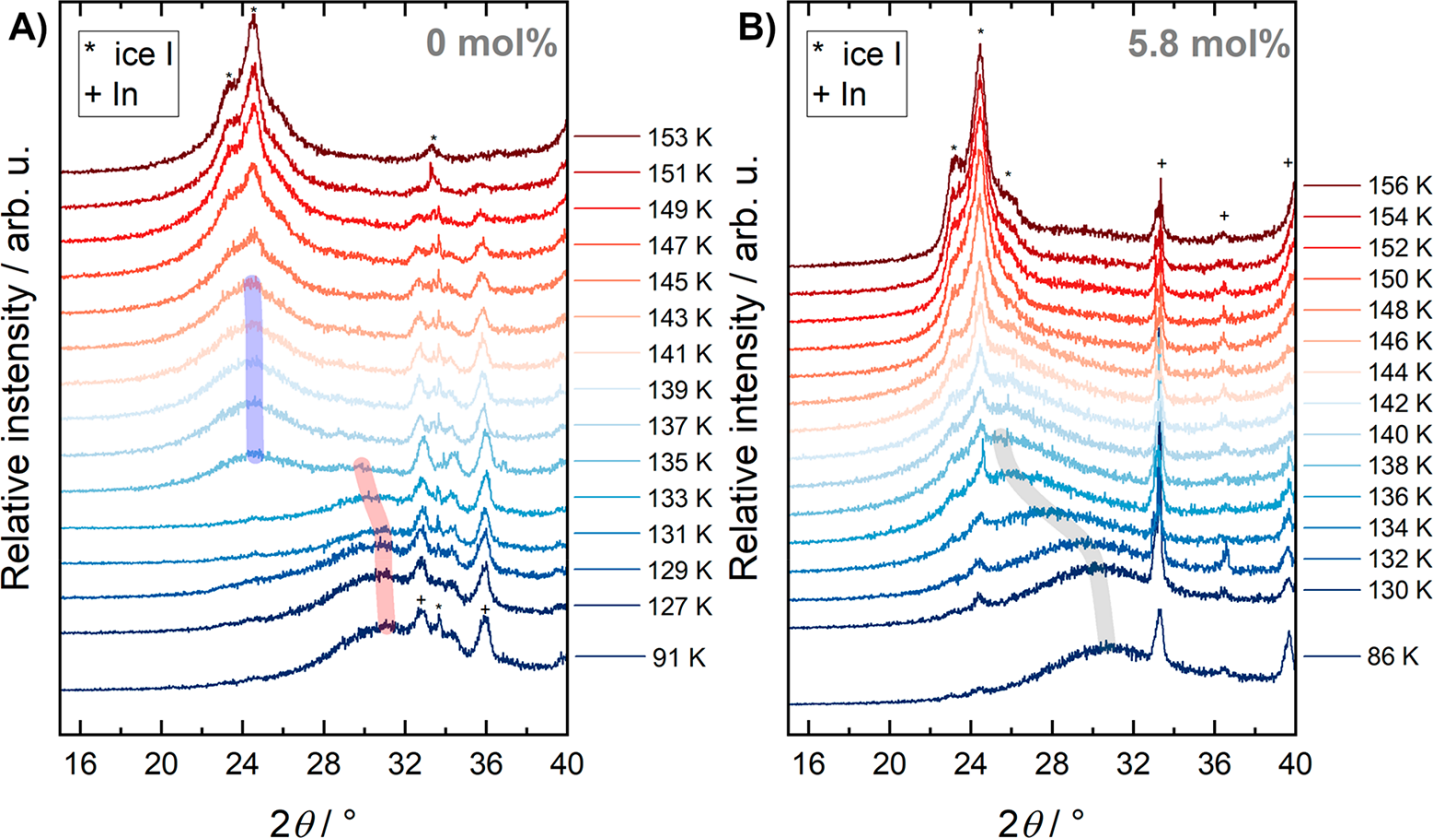
X-ray diffractograms of (A) compressed pure HGW (HDA, dHGW) and (B) compressed hyperquenched aqueous solution with 5.8 mol % (*R* = 16.2) LiCl (LiCl-HDA) during heating. The maxima of the halo peaks are indicated by transparent guide lines. Bragg peaks of ice I and indium are marked by asterisks and plusses, respectively. The curves are stacked vertically for clarity according to the temperature at which the measurement was performed. The indicated temperatures were corrected for thermal contact using the DSC data.

After compression, the halo-peak maximum shifts significantly, in the case of the 5.8 mol % (*R* = 16.2) sample from 25.7° ± 0.2° to 30.9 ± 0.2° (see Figure 2B). This is very close to the values reported for pure water HDA,^4,13,46^ from which we infer that after compression, a glass of higher density is obtained. This can be explained using two approaches: (i) discontinuous LiCl-LDA → LiCl-HDA transition due to the polyamorphic behavior that was suggested in the dilatometric experiment of the 5.8 mol % sample, and (ii) continuous densification unrelated to polyamorphism. We now attempt to distinguish the two scenarios by examining X-ray diffractograms of pure water HDA and compressed hyperquenched LiCl solution with 5.8 mol % in the course of stepwise heating at subambient pressure (Figure 3). The idea behind this experiment is that all densified ices transform back to low-density ice at subambient pressure, namely, once the temperature is high enough for sufficient mobility of oxygen atoms to cross the activation barrier. That is, all HDA-like parts in the sample will eventually transform to LDA and later to ice I. The question is whether this transition occurs continuously in a broad temperature range or discontinuously at one single, specific temperature.

First, we focus on the observations made for pure water: The maximum of the halo-peak is observed at 31.2° ± 0.2° at 91 K, typical of HDA.^13,46^ Upon heating, the halo-maximum shifts to slightly lower values (indicated by the red guide to the eye in Figure 3A), but remains ∼30° up to 133 K. At 135 K, the halo peak position jumps to 24.7° ± 0.2° (maximum indicated by a blue guide to the eye in Figure 3A). This abrupt shift makes the case for the polyamorphic transition of HDA to LDA. At 135 K, there is still a small amount of the 30° halo left, making the case for two distinct glasses being present simultaneously (see Figure S2 in the Supporting Information). This implies that, out of the first state (HDA), a second one (LDA) emerges and hints at a mechanism involving nucleation of LDA and subsequent growth at the expense of the HDA matrix. At 137 K and above, only the halo peak at 24.7° ± 0.2° is observed, which barely shifts upon heating. That is, the single transition temperature is identified as 135 K. Similar observations would also be expected for the 5.8 mol % (*R* = 16.2) LiCl solution if scenario (i) as presented above applies. In the literature, a similar case was observed only upon quenching a sample halfway through the decompression-induced polyamorphic HDA → LDA transition.^45^ In this case, the chemical potentials of LDA and HDA are almost equal (within hysteresis) at the transition and coexistence is observed. Here, we transform quenched HDA by heating at low pressure. Under these conditions, the chemical potential of LDA is much lower than the one of HDA, i.e., no coexistence in the thermodynamic sense is expected. That is, the double halo peak at 135 K is a result of limited kinetics, possibly even small thermal gradients. Nonetheless, we are speaking about one glass emerging out of a second glass of different density.

An equivalent diffraction protocol was carried out for the 5.8 mol % (*R* = 16.2) sample, as shown in Figure 3B. Upon heating, the halo peak shifts smoothly from 30.8° ± 0.2° to lower values (indicated by the gray guide to the eye) in the range between 130 K and 140 K, without any specific temperature, at which a new glass appears. This is in stark contrast to the case of pure water HDA, in which no intermediate states are encountered and the halo peak shifts abruptly at the polyamorphic transition. That is, the transition seems to have a continuous character, unlike the polyamorphic transition. It can be interpreted in favor of scenario (ii) as a sign of gradual structural relaxation of the high-pressure equilibrated glass toward its preferred glass configuration at ambient pressure. A similar case was found using Raman spectroscopy, albeit for a solution with ∼9 mol % LiCl (*R* ≈ 10), which is in the salt-rich domain, where solutions vitrify easily.^28^ However, we stress that this alone is not decisive proof for a truly continuous transformation. Also, a case in which both a continuous and a sharp transition occur might explain the data in Figure 3B. As discussed above, we observe a large increase in fwhm with increasing molar fraction of LiCl. At 134 K in Figure 1B, the halo is even broader than that observed at 80 K in Figure 2A and might be composed of two broad halo peaks. As soon as two broad halo peaks begin to overlap, they are harder to identify unambiguously. In the end, it is unclear whether one, two, or even three halos are at the origin of the diffractograms in Figure 2B at 130–137 K.

At 138 K, a peak maximum of 25.8° ± 0.2° is obtained, which implies that the high-density glass has fully reverted to the initial state of lower density. Based on this seemingly continuous transformation behavior, it is not justified to call this state (nor the hyperquenched material of the same composition) LDA. Already at 5.8 mol %, the solution can be regarded as a concentrated solution (CS). XRD alone also does not allow us to deduce whether a similar ambiguity also applies to our other, more dilute solutions. To resolve this ambiguity we have resorted to calorimetry, where we present our analysis in the following section. By contrast to the volumetric study presented in Figure 1 and the X-ray study summarized in Figures 2 and 3, the calorimetry study allows us to access also information related to dynamics, e.g., glass transition temperatures and structural relaxation times. Mixtures of two or more components can be identified in thermograms based on their distinct behavior, e.g., distinct transition temperatures.

### 3.3 Differential Scanning Calorimetry

#### 3.3.1 Polyamorphism

**Figure 4:**
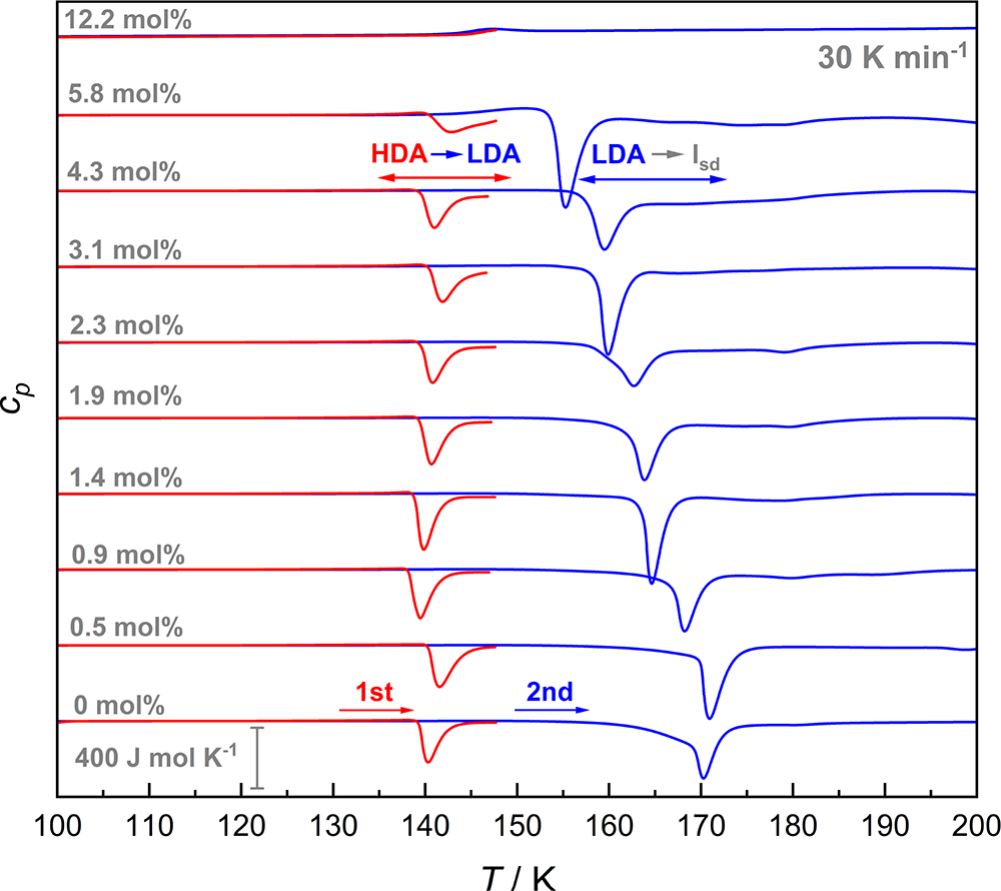
First (red) and second (blue) heating trace (30 K min^–1^) of the quench-recovered LiCl-HDA samples made from LiCl-HGW (0–5.8 mol %, *R* = ∞–16.2). The first heating scan shows the thermal behavior of LiCl-HDA while the second one (obtained after recooling from ∼148 K) corresponds to the one of LiCl-LDA. For comparison, the thermograms of compressed CS (12.2 mol %, *R* = 7.2) are included. The traces are normalized to the moles of water as described in the Methods section and shifted for clarity.

Figure 4 shows calorimetry traces obtained by heating the compressed samples at 30 K min^–1^. The scans for the other heating rates (10, 20, 40, and 50 K min^–1^) can be found in the Supporting Information. Between 0 and 4.3 mol % (*R* = ∞–22.3), we observe a pronounced exotherm with an onset temperature between 139 and 141 K (*T*_Poly_) in all first heating scans (red lines in Figure 4). For pure water and pressure-vitrified LiCl solutions, this feature was assigned to the polyamorphic transition from high-density glass to low-density glass.^5,23^ This is consistent with the dilatometry and XRD results discussed above. In the second scan, we reheat the low-density glasses (blue lines in Figure 4) and observe another pronounced exotherm with an onset temperature between 154 K and 170 K (*T*_*x*_). The X-ray diffractograms in Figures 3A and 3B show that Bragg peaks of ice I develop above 150 K, most notably the characteristic Bragg peak at 24°. That is, we assign this exotherm to the cold-crystallization of LDA to ice I, which is well-known to occur in this temperature range.^47^

The densified solution with 5.8 mol % (*R* = 16.2) shows a quite similar thermal behavior, but it is evident to the naked eye that the first exothermic peak (red trace) has lost its typical shape and is unusually broad. Such a non-Gaussian shape of exotherms is typical of continuous relaxation processes. This is in agreement with the continuous structural relaxation observed in diffraction (see Figure 3B), but not with a genuine polyamorphic transition. The relaxed glass then cold-crystallizes in the second heating scan. The reference CS (12.2 mol %, *R* = 7.2) shows neither a polyamorphic transition nor a cold-crystallization event. Instead, only a reversible glass transition with an onset temperature *T*_g,CS_ of 144 ± 1 K (first heating) or 142 ± 1 K (second heating) is observed. From this, we confirm that also the CS does not show any polyamorphism as noted in the literature.^23^ We emphasize that our high-pressure annealing protocol specifically designed for HDA relaxation does not seem to have a notable effect on a CS of this composition.

**Figure 5:**
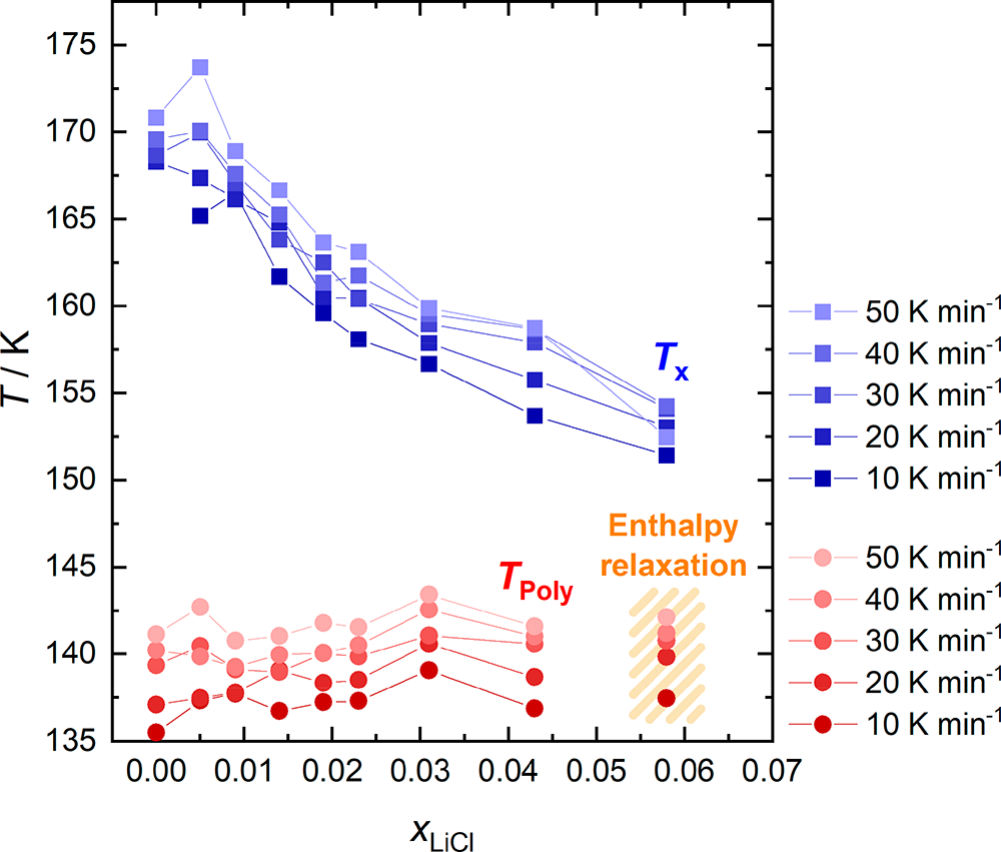
Onset temperatures of the polyamorphic transition (*T*_Poly_) and the cold-crystallization (*T*_*x*_) to ice I, as a function of the mole fraction of LiCl (*x*_LiCl_) for heating rates between 10 and 50 K min^–1^. In the case of the 5.8 mol % solution, it is no longer a polyamorphic transition, but structural relaxation of the glass, as discussed in the main text.

All onset temperatures of the polyamorphic transition (*T*_Poly_) and the cold-crystallization (*T*_*x*_) are shown in Figure 5, as a function of mole fraction *x*_LiCl_. Both transformation temperatures are sensitive to heating rates, where higher ones generally lead to higher transition temperatures. Notably, *T*_Poly_ is hardly influenced by *x*_LiCl_ and located at ∼136 K for the lowest and ∼142 K for the highest heating rate. This signifies that the thermal stability of our HDA is not affected by the Li^+^ and Cl^–^ ions. Rather, it is solely governed by the high-pressure annealing procedure.

On the other hand, *T*_*x*_ shows contrasting behavior with increasing mole fraction. While pure LDA transforms to ice I at ∼170 K, LiCl-LDA cold-crystallizes at temperatures as low as 155 K at 4.3 mol % (*R* = 22.3). Please note the significant fronting of the cold-crystallization exotherms in Figure 4. These asymmetric peaks imply that the initial temperature at which cold-crystallization commences is significantly lower than the onset temperatures given in Figure 5. When referring to both initial and onset temperatures, the same trend is observed: The presence of LiCl accelerates LDA crystallization so that *T*_*x*_ is lowered. This is surprising for two reasons: (i) adding LiCl usually hinders crystallization, as seen in the example of ice freezing from the liquid solution, and (ii) it was found that the presence of LDA domains is a prerequisite for the formation of ice I.^36,48^ The significant lowering of *T*_*x*_ with *x*_LiCl_ implies that LiCl plays a role, not only pure H_2_O-LDA domains. For a phase-separated glass consisting of pure LDA domains and CS domains, we would anticipate hardly any impact on *T*_*x*_. That is, the phase separation hypothesis is inconsistent with our observation. The hypothesis of a homogeneous distribution of LiCl in LDA, on the other hand, allows one to explain this effect: The presence of ions distorts the tetrahedral LDA network toward a more HDA-like character and thereby supports the formation of ice I. That is to say, LDA is indeed the mother of ice I, but LDA modified through ions nearby is even more efficient. In terms of thermodynamics, the LDA structure is destabilized by adding the salt, whereas ice I remains unaffected due to the insolubility of the salt in ice I.^49,50^ In accordance with the Hammond postulate,^51^ destabilization of LDA then also lowers the activation barrier to reach the transition state and accelerates cold-crystallization. The destabilization is both due to an enthalpic effect and entropic effect where a structurally distorted and higher-entropy state is reached by adding the salt.

#### 3.3.2 Glass Transitions

**Figure 6:**
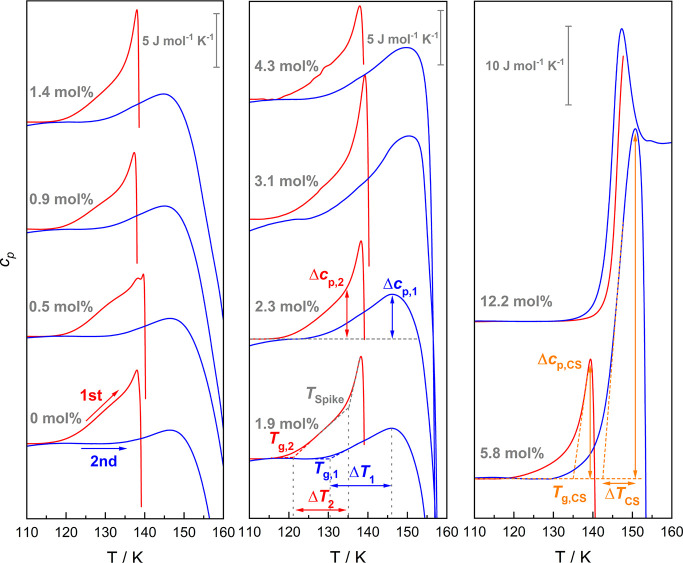
Magnification of the traces in Figure 4 with a focus on the glass-to-liquid transitions. *T*_g,2_ and *T*_g,1_ marked for the 1.9 mol % scan are the glass transition temperatures of LiCl-HDA (red) and LiCl-LDA (blue), respectively. *T*_Spike_ is the onset temperature of the spike-like feature that commences right after *T*_g,2_. *T*_g,CS_ corresponds to the glass transition temperature of a concentrated solution and is only observed at ≥5.8 mol % (*R* < 16.2). The definitions for the change in heat capacity (Δ*c*_p,2_ (LiCl-HDA), Δ*c*_p,1_ (LiCl-LDA), and Δ*c*_p,CS_ (CS) are marked in the traces of the 2.3 mol % and the 5.8 mol % sample, respectively. The width of the glass transition Δ*T*_2_ (LiCl-HDA), Δ*T*_1_ (LiCl-LDA), and Δ*T*_CS_ (CS) is defined as indicated for the 1.9 mol % and 5.8 mol % samples.

**Figure 7:**
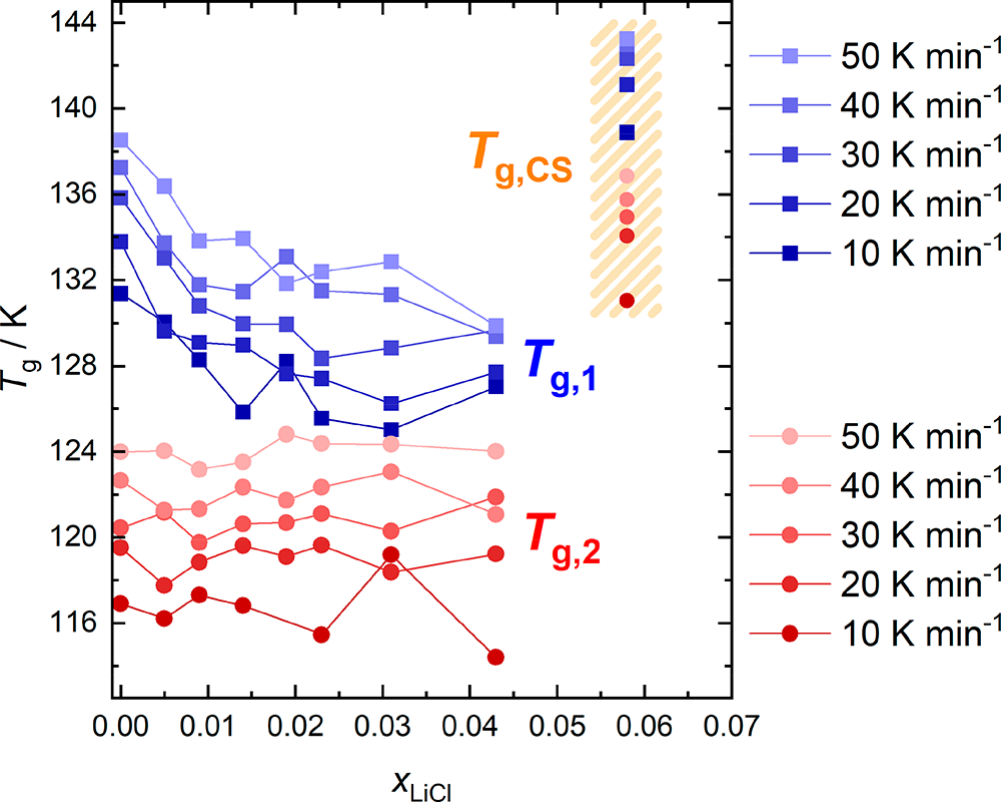
Glass-transition temperatures of LiCl-HDA (*T*_g,2_), LiCl-LDA (*T*_g,1_), and CS (*T*_g,CS_), as a function of mole fraction of LiCl (*x*_LiCl_) for different heating rates.

A magnification of Figure 4 in the temperature range near *T*_*poly*_ and *T*_*x*_ is provided in Figure 6 (note the two different scale bars in the figures). For pure water HDA (0 mol % red curve), we observe an increase of heat capacity at 120 ± 1 K and another one at 136 ± 1 K in the two heating scans. The first increase (red curves in Figure 6) is identified as the second glass transition of water (*T*_g,2_), namely, the transition of HDA to HDL that first was identified by Winkel et al. at 116 K.^11^ The difference between this and our value is the heating rate, where Winkel et al. used a rate of 10 K min^–1^, but Figure 6 shows data for 30 K min^–1^. For a heating rate of 10 K min^–1^, we also observe a transition at 116 K, as summarized in Figure 7. The second increase at ∼135 K, also called a *spike*, was recently suggested by us to be caused by the nucleation barrier that must be overcome before the growth of low-density water occurs within HDL.^14^ After the spike, the growth of bulk low-density water releases the energy at the origin of the polyamorphic transition. This is indicated by the massive exotherm that extends beyond the zoom level of the *y*-axis in Figure 6. As is evident from Figures 6 and 7, no pronounced shifts in the onset of the spike and *T*_g,2_ are observed upon increase of *x*_LiCl_. The phenomenology changes for *x*_LiCl_ ≥ 5.8 mol % (*R* < 16.2): There is no longer an initial heat capacity increase of the LiCl-HDA glass transition followed by a spike. Instead, there is just a single but much larger increase in heat capacity. This increase is immediately followed by a sharp enthalpy relaxation for the 5.8 mol % solution. Please note the much sharper drop from the highest point in *c*_p_ for 5.8 and 12.2 mol % in Figure 7, compared to all other solutions. The more edge-like feature is reminiscent of the overshoot effect typically seen for all “simple” glass transitions^15,27^ and is associated with sudden and fast relaxation. That is, this represents a “simple” glass transition for the concentrated solution without the complication of the polyamorphic transition. It is observed here at an onset temperature of *T*_g,CS_ = 142 ± 1 K, both for the reference CS at 12.2 mol % and the hyperquenched and densified solution at 5.8 mol %. For more dilute solutions, the glass transition shows the spike but a more rounded, slower release of enthalpy. Both features are hallmarks of the polyamorphic transition, only observed for this type of transition. The high-pressure-equilibrated glass first experiences a glass-to-liquid transition. Only in the deeply supercooled liquid, the water molecules in the solution can rearrange to the more favored low-density state, due to their increased mobility.

The second heating scan of pure water LDA (0 mol % blue curve in Figure 6) shows a more feeble increase in heat capacity just before the cold-crystallization to ice I commences. This feature corresponds to the glass-to-liquid transition of LDA *T*_g,1_.^52−54^ At 30 K min^–1^, we determine *T*_g,1_ = 136 ± 1 K (see Figure 7), which is in excellent agreement with the value reported in the literature.^52^ Upon addition of LiCl, *T*_g,1_ first decreases (from 136 ± 1 K to 128 ± 1 K between 0 and 2.5 mol % for 30 K min^–1^) and exhibits a broad minimum between 2.5 and 4.3 mol % (*R* = 40.7–22.3). These results demonstrate that LiCl has a much stronger influence on *T*_g,1_ of LDA than on *T*_g,2_ of HDA. The glass transition temperatures are governed by the dynamics^55^ of LDL and HDL, which are, in turn, dictated by the structure of their hydrogen-bonded networks.^56^ This implies that the LDA network is much more sensitive to the electrostatic forces exerted by the ions than the HDA network.

At 5.8 mol %, the behavior again changes as *T*_*g*_ increases and even reaches a value of ∼140 K, significantly above the *T*_g,1_ of pure LDA. In addition, the heat capacity increase for the 5.8 mol % solution is no longer feeble, but much larger (see Figure 9). It no longer bears any resemblance to the subtle glass transition observed in pure LDA. This is because, after the glass has been equilibrated at low pressure, it behaves similarly to CS. The only difference to the reference CS is the tendency to cold-crystallize, which is due to the significantly higher water content.

**Figure 8:**
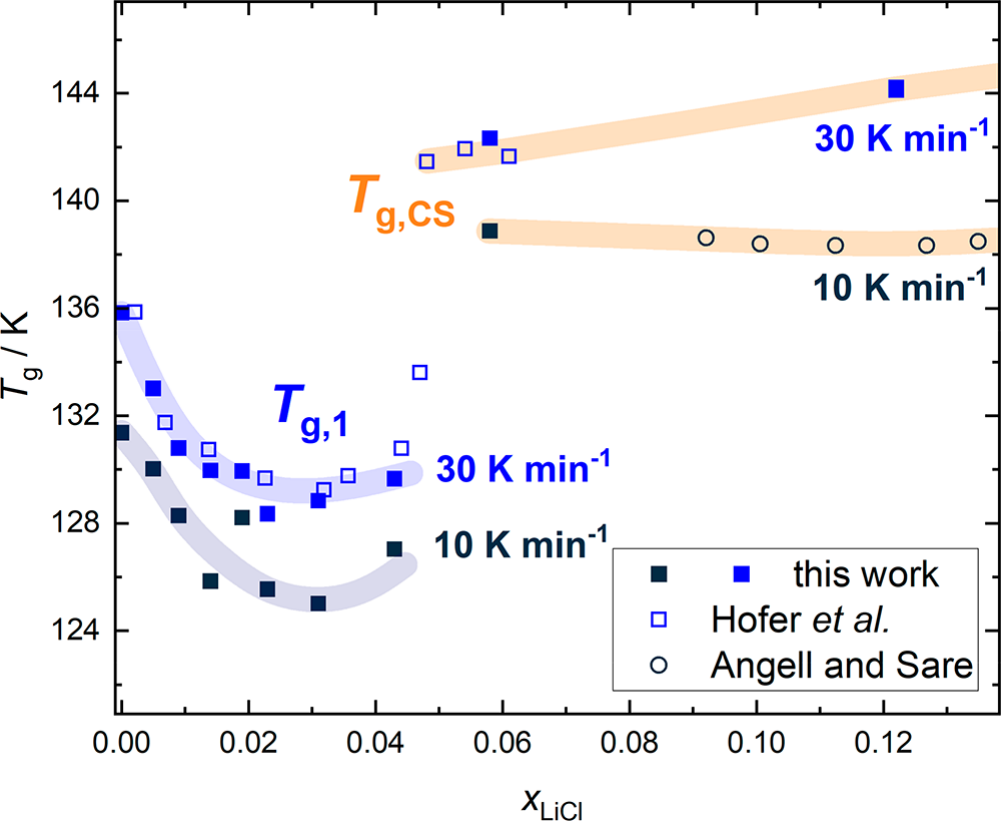
Comparison of *T*_g,1_ with literature data^15,17^ for heating rates of 30 and 10 K min^–1^. Hofer et al.^17^ used a heating rate of 30 K min^–1^, whereas Angell and Sare employed a heating rate of 7–9 K min^–1^.^15^ We consider the latter to be comparable to 10 K min^–1^. Broad transparent lines serve as guides to the eye. Values that correspond to concentrated solutions are labeled *T*_g,CS_ and marked orange.

In Figure 8, we compare our data of *T*_g,1_ of LiCl-LDA (made from HGW via LiCl-HDA) with existing literature data on LiCl-HGW (not obtained via LiCl-HDA)^17^ and on LiCl-CS.^15^ The low-concentration region was sampled in ref (17) with a heating rate of 30 K min^–1^. Our *T*_g,1_ values for LiCl-LDA (obtained after heating LiCl-HDA) agree very well with the data on LiCl-HGW. The initial decrease, the broad minimum and also the steep increase at ∼5 mol % (*R* ≈ 19) are reproduced, although the thermal history is quite different for both glasses. Most importantly, in one dataset, the polyamorphic transition was observed (our data here), whereas in the other dataset, it was not (Hofer et al.^17^ data). The exact match of glass transition temperatures is surprising and denies the possibility of a phase separation occurring upon hyperquenching. It is contradicting the idea of a forced polyamorphic phase separation of LiCl-HDA into pure LDA and CS upon the hyperquenching voiced by Suzuki,^23^ at least at the rates of 10^7^ K s^–1^. If such a phase separation occurred, *T*_g,1_ would be unaffected and would remain at ∼136 K, independent of the mole fraction of LiCl. That is, when starting from hyperquenched solutions, LiCl ions are homogeneously dispersed in both LDA and HDA, before and after the polyamorphic transition. If there was phase separation, it must occur already upon hyperquenching and persist even in the high-density state.

The concentrated solutions were measured with a heating rate of 7–9 K min^–1^ in ref (15), which is similar to the 10 K min^–1^ employed by us. Here, we find that the *T*_g_ value of the 5.8 mol % (*R* = 16.2) solution is identical with *T*_g,CS_ up to ∼14 mol % (*R* = 6.1), which reinforces the idea that the 5.8 mol % glass belongs to the family of CS. That is, the steep increase of *T*_g,1_ is in fact due to a switchover from *T*_g,1_ to *T*_g,CS_ and marks the end of water polyamorphism. In other words, it signifies the change from water-dominated to solute-dominated behavior in LiCl–water.

**Figure 9:**
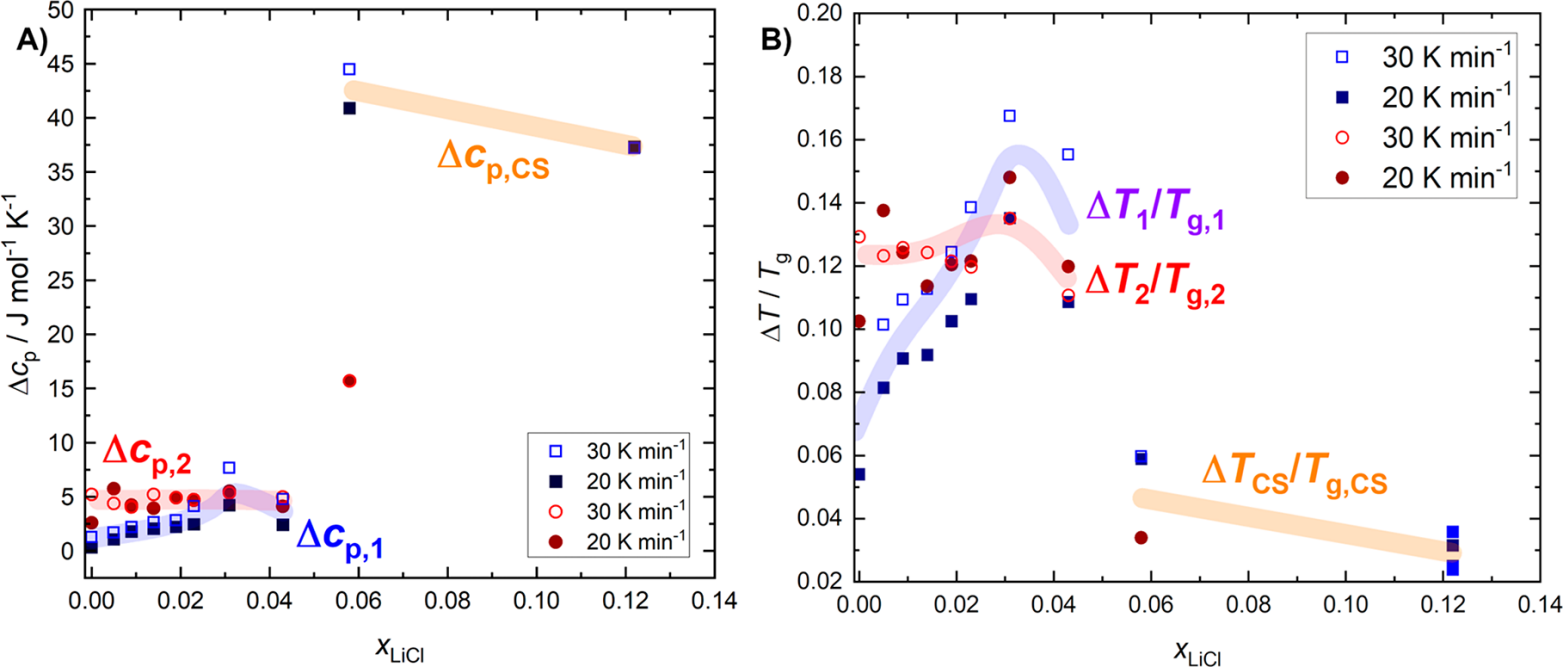
(A) Change in heat capacity (Δ*c*_p_) per mole of water in the sample and (B) relative width of the glass transition Δ*T*/*T*_g_ as a function of *x*_LiCl_. Broad transparent lines serve as a guide to the eye. The observed trends are similar for all heating rates (see the Supporting Information) but, for simplicity, only the results for 30 and 20 K min^–1^ are shown. The definitions of Δ*c*_p_ and Δ*T*/*T*_g_ are shown in Figure 6. Textbook definitions of Δ*c*_p_ and Δ*T*/*T*_g_ are not useful for our DSC traces, since no unambiguous end point could be determined due to the exothermic features (polyamorphic transition or cold crystallization) commencing right after the glass transitions. For consistency, the same procedures were applied to the CS. The error estimated for Δ*c*_p_ is roughly 0.4 J mol^–1^ K^–1^ and that for Δ*T*/*T*_g_ is ∼0.01. Error bars are omitted for clarity.

These assessments are further backed upon examining the change in heat capacity *Δc*_*p*_ and the relative width of the glass transition *ΔT/T*_g_ as a function of *x*_LiCl_ (Figure 9). In particular, both metrics show a behavior comparable to the glass transition temperatures: For LiCl-HDA, no influence of *x*_LiCl_ on *Δc*_*p*_ is found for any heating rate. The *Δc*_*p*_ of the glass transition of LiCl-LDA however, shows a slight monotonous increase between 0 and 4.3 mol % (R = ∞-22.3) before suddenly jumping to values that are ∼10 times larger at 5.8 mol % (R = 16.2). This indicates the change from water- to solute-dominated regime and is corroborated by the similar *Δc*_p_ of the 12.2 mol % (R = 7.2) CS.

For the Δ*T*_2_/*T*_g,2_ of LiCl-HDA, we observe no clear trend with increasing mole fraction. However, we note that the evaluation of Δ*T*_2_/*T*_g,2_ of LiCl-HDA is rather error-prone, because the spike gets more pronounced upon concentration increase, and thus, makes it more difficult to determine the end point of *T*_g,2_. For LiCl-LDA, we determine a continuous increase of Δ*T*_1_/*T*_g,1_ with added LiCl until a sudden decrease at 5.8 mol % signals entering the CS domain. Both metrics in Figure 9 can be used to assess the fragility of the supercooled liquid: broad (large Δ*T*/*T*_g_) and feeble glass transitions (small Δ*c*_p_) are typical of strong liquids. HDL was identified as a strong liquid, and LDL even as a superstrong liquid in earlier work.^11^

The strong nature of HDL is barely affected by the addition of salt, whereas the glass transition in LDL gets even broader, but with a larger increase in heat capacity. From these observations, it is ambiguous how fragility is affected by the presence of ions. This is especially so because the end point of the glass transition is not seen as it is superposed with the heat released at the polyamorphic transition. That is, a different analysis to make statements about the fragility of salty LDL is necessary in the future.

#### 3.3.3 Calorimetric Relaxation Times

From the heating rate dependency of *T*_g,1_, *T*_g,2_, and *T*_g,CS_, we extract activation energies at the glass transition *E*_*g*_ under the assumption that it can be described by an Arrhenius equation,^57^
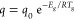
1where *q* is the heating rate, *q*_0_ the pre-exponential factor, and *R* the gas constant. The use of an Arrhenius ansatz is justified based on the strong and superstrong nature of HDL and LDL, respectively, as noted above. After fitting a linearized form of eq 1 to our data, we obtain *E*_g_ from the slope. The calculated values for *E*_g_ are shown in the Supporting Information (see Table S1 in the Supporting Information). Using *E*_g_, *q*, and *T*_g_, we further calculate calorimetric relaxation times (τ_cal_) for each composition at the corresponding glass transition temperature *T*_g_) (via eq 2.^11,58^
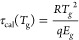
2

The values for τ_cal_(*T*_g_) are listed in the Supporting Information (see Table S1). The temperature dependency of the relaxation time can be expressed in terms of eq 3, again given that it follows Arrhenius behavior:^57^
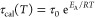
3where τ_0_ is a pre-exponential time constant and *E*_A_ is the activation energy. At *T*_g_, τ_cal_(*T*_g_) can be written as
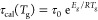
4

Assuming that τ_0_ and *E*_A_ are temperature-independent and that *E*_A_ = *E*_g_, one can combine eqs 3 and 4 to write the following expression:
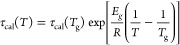
5

**Figure 10:**
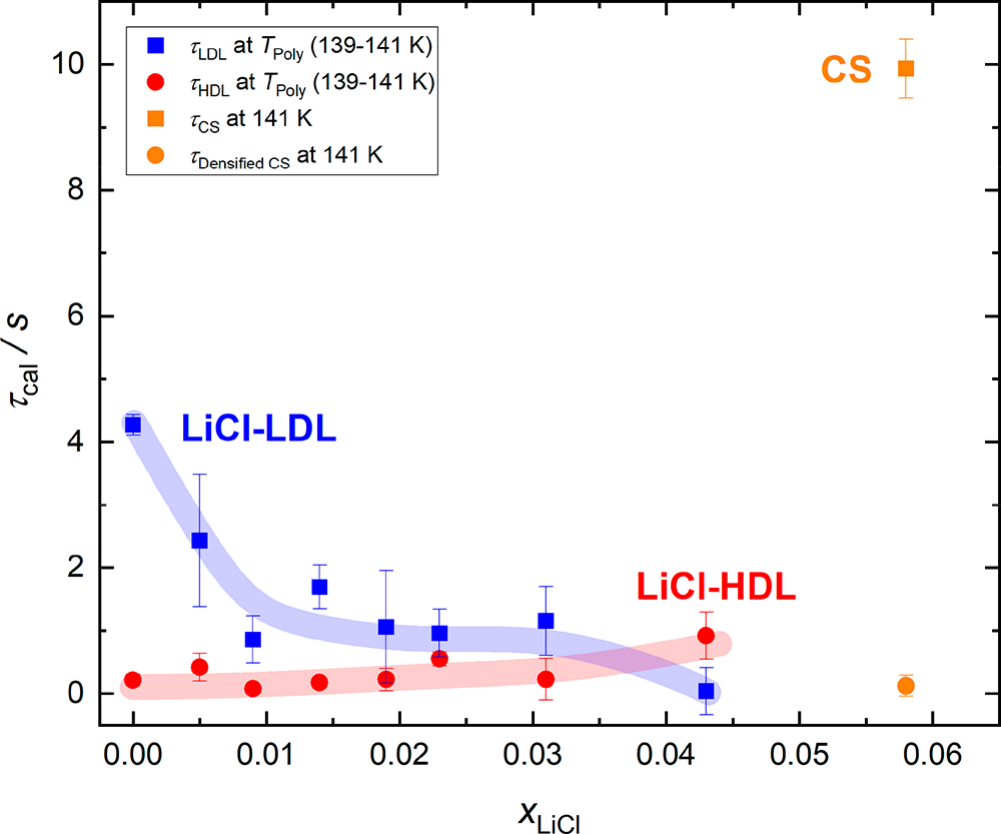
Calorimetric relaxation times τ_cal_ estimated from the heating rate dependency of *T*_g,1_, *T*_g,2_, and *T*_g,CS_ via eqs 1–5. Error bars indicate the estimated error based on the error of *E*_g_ that is obtained from the corresponding fits of eq 1 to our data. The relaxation times for LDL and HDL are shown at *T*_Poly_ measured at 30 K min^–1^ (139–141 K). The relaxation times of CS are shown at the *T*_Onset_ of the enthalpy relaxation at 30 K min^–1^ (141 K). Broad transparent lines serve as guides to the eye. τ_HDL_ and τ_LDL_ are the calorimetric relaxation times of LiCl-HDL and LiCl-LDL respectively. τ_Densified CS_ and τ_CS_ correspond to a (densified) CS and are highlighted in orange color.

The values of τ_cal_ at *T*_Poly_ for LiCl-HDL (τ_HDL_) and LiCl-LDL (τ_LDL_) calculated using eq 5 are shown in Figure 10. Please note that, for *T*_Poly_, we used the values that we determined for 30 K min^–1^ (see sections 3.3.1 and 3.3.2). *T*_Poly_ is the same for both, LiCl-HDL/LDL, and assumes values between 139 and 141 K. For the relaxation time of the CS τ_CS_ (or τ_Densified CS_ for the densified CS), we used the *T*_Onset_ of the enthalpy relaxation instead, which is found at 141 K. In general, all calorimetric relaxation times range between 0.1 s and 4 s in Figure 10. That is, all relaxation times are well below the definition criterion of 100 s that delineates glassy solids from ultraviscous liquids. Both LDL and HDL indeed must be regarded as ultraviscous liquids according to the relaxation times deduced from the rate dependence of the glass transition in calorimetry. It is evident that τ_HDL_ shows only a small concentration dependency and remains below 1 s between 0 and 4.3 mol % (*R* = ∞–22.3). This indicates that LiCl barely influences the relaxation dynamics of HDL as one would expect based on the nearly absent concentration dependency of *T*_g,2_. Even τ_Densified CS_ at 5.8 mol % remains in the range of τ_HDL_. In other words, the relaxation dynamics of the densified CS is similar to the dynamics in (LiCl-)HDL. This corroborates the idea that LiCl has little impact on the dynamics of HDL.

LiCl-LDL behaves differently: Between 0 and 4.3 mol %, τ_LDL_ decreases from ∼4 s to <1 s. This fits the trend observed for *T*_g,1_ very well as lower relaxation times correspond to lower glass transition temperatures. This supports the idea of the plasticizing effect of LiCl on the hydrogen-bonded LDA/LDL-network,^17^ which is then responsible for lowering *T*_g,1_ and τ_LDL_. τ_CS_ at 5.8 mol %, on the other hand, is located at considerably larger values than τ_LDL_. Thus, the CS has comparably slower relaxation dynamics, leading to an increase of the glass-transition temperature. Thus, there is a pronounced and sharp change in the relaxation dynamics of LDL between 4.3 and 5.8 mol % that indicates the departure from water to solute-dominated behavior. For HDL such a sudden shift is missing. In section 3.3.2, we inferred that the CS (at 5.8 mol %) is structurally more HDA-like, based on the shift of the halo peak maximum. A similar hydrogen-bond network, and thus a similar structure, would imply similar relaxation dynamics of HDL and the densified CS. Our estimated values of τ_HDL_ and τ_Densified CS_ suggest that this is the case.

In terms of activation energies, we deduced 34 kJ mol^–1^ both for LDL and HDL based on dielectric relaxation spectroscopy in a preceding work.^11^ This is reasonably close to the values of 33.7 kJ mol^–1^ for LDL and 27.3 kJ mol^–1^ for HDL deduced in this work via calorimetry (see Table S7). Thus, the activation process at the origin of the dielectric loss seems to be the same as the one relevant for the shift in calorimetric glass transition temperatures. These activation energies correspond roughly to the enthalpy needed to break a single hydrogen bond, which is necessary for translational motion of water molecules at the heart of the relaxation. By contrast, orientational motions (without translational motions) are associated with much higher activation energies, e.g., 81 kJ mol^–1^ for the rotational motion of water molecules in the ice V crystal. After adding salt, the activation energies for relaxation stay roughly constant, in the range between 27 kJ mol^–1^ and 39 kJ mol^–1^ for LiCl-LDL and between 20 kJ mol^–1^ and 32 kJ mol^–1^ for LiCl-HDL. With a typical error bar of ±5 kJ mol^–1^ (see Table S1), a significant trend cannot be inferred. However, in the case of the CS, the activation energies are in the range of 60–90 kJ mol^–1^, which is a factor of 2–3 higher. This again underlines that the arrangement of molecules and ions in the CS is very different from the one in LiCl-LDL and LiCl-HDL.

## 4 Discussion and Conclusion

We here present a comprehensive study related to the impact of LiCl on the phase behavior of noncrystalline water below 180 K. In this range, the polyamorphic transition between two types of amorphous water is the most interesting phenomenon that is discussed as the low-temperature equivalent of a liquid–liquid transition. The latter might end in a virtual liquid–liquid critical point, hidden behind the curtain of crystallization. These phenomena might be at the origin of many anomalies of water in the supercooled and deeply supercooled state. In our study, we combine volumetric, calorimetric, and diffraction observations to provide a comprehensive picture. We use the technique of hyperquenching at ∼10^7^ K s^–1^ to vitrify millions of micrometer-sized liquid droplets, which is necessary to avoid crystallization before vitrification. Previously, the dilute solutions studied here were regarded as crystal-forming, i.e., impossible to reach the glassy state through cooling of the liquid. Furthermore, we transfer 200–400 mg of vitrified material to our high-pressure setup in order to densify it, more specifically to turn it from the low-density glass (LDA-LiCl) into the high-density glass (HDA-LiCl). Also, this is a novel approach in the literature, where we access the polyamorphic transition, not starting from crystals, but from the liquid phase.

Our observations point toward the suppression of polyamorphic behavior of water in pressurized hyperquenched solutions at *x*_LiCl_ ≥ 5.8 mol % (*R* = 16.2). This is at considerably lower mole fractions than in the case of glycerol–water (*x* ≈ 10–15 mol %, *R* ≈ 9–6)^21,22,59^ and most other polyol solutions.^60^ Interestingly, it is also below the end of polyamorphism as estimated for pressure-vitrified LiCl solutions (*x* ≈ 10 mol %, *R* ≈ 9).^23^ However, the water-rich, but non-polyamorphic densified solution of 5.8 mol % shows unusual traits that, at first glance, feign polyamorphic behavior. This includes (i) a shift in halo peak maximum from 2θ ≈ 30° to ∼25° in XRD analysis and (ii) an exothermic transition at temperatures close to *T*_poly_ in calorigrams. However, closer inspection shows a continuous change in halo position with temperature rather than the jumplike nature known for the polyamorphic transition. The exothermic transition turns out to be related to structure relaxation rather than to the polyamorphic transition: the activation energies extracted from rate-dependent calorimetry are higher by a factor of 3, compared to the activation energy in both LDL and HDL.

**Figure 11:**
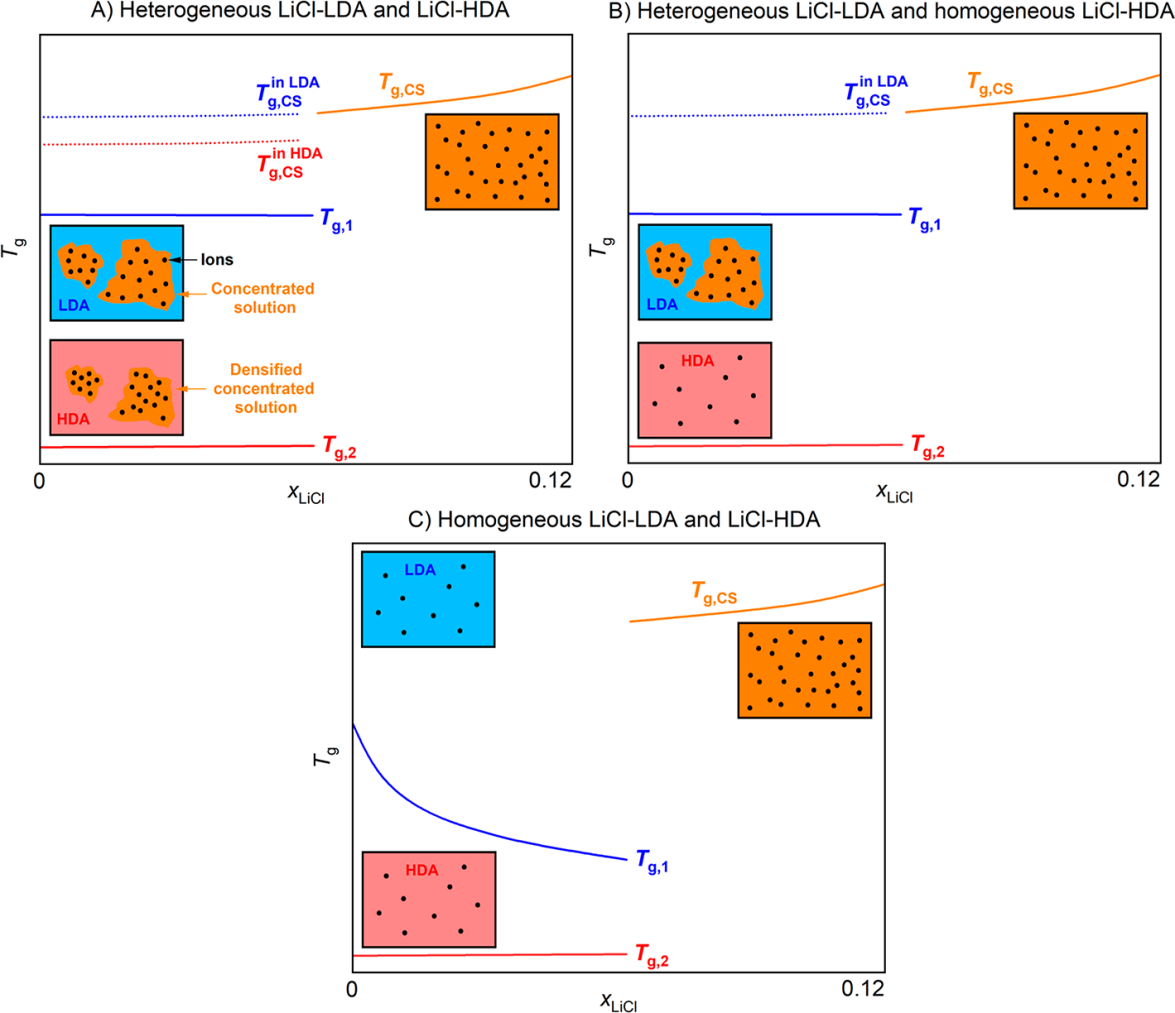
Three possible scenarios regarding the behavior of glassy LiCl_aq_ and the hypothesized effects on the glass transition temperatures based on literature and our results. (A) Both LiCl-LDA and LiCl-HDA are heterogeneous and phase separate into a water-rich part (LDA or HDA) and a CS. At low concentrations, this results in the appearance of the glass transitions of pure HDA *T*_g,2_ or LDA *T*_g,1_, along with the glass transition of the separated CS in the HDA (*T*_g,CS_^in HDA^) or LDA (*T*_g,CS_^in LDA^) matrix. At high concentrations (≥5.8 mol %, *R* = 16.2), only the glass transition of the CS *T*_g,CS_ is observed. (B) LiCl is only insoluble in LDA but not in HDA. Therefore, HDA is homogeneous and separates into LDA and a CS in the course of the polyamorphic transition. In contrast to the scenario described in panel (A), no *T*_g,CS_^in HDA^ is observed. (C) LiCl-LDA and LiCl-HDA are fully homogeneous. Below 5.8 mol %, either *T*_g,2_ (LiCl-HDA) or *T*_g,1_ (LiCl-LDA) appears, whereas above 5.8 mol %, neither LiCl-HDA nor LiCL-LDA are observed but a homogeneous CS is formed instead. Only the *T*_g,CS_ value is found at ∼140 K, so that a jumplike change in *T*_g_ is found at ∼5 mol %.

Within the polyamorphic regime below 5.8 mol %, we determined the glass transition temperatures of both LDA and HDA upon heating at ambient pressure and temperature-dependent relaxation times. In order to understand the impact of LiCl on water’s two glass transitions, we discuss the three possible scenarios described in Figures 11A–C that were implied in previous works (sketched in Figure 11). Figure 11A shows a phase separation into solute-rich and solute-poor regions, regardless of whether the water is in a low-density state or a high-density state. LiCl mixes neither with LDA nor HDA, and a concentrated solution CS that is neither LDA- nor HDL-like must be formed to accommodate the ions. This view receives support from calorimetry and Raman experiments on pressure-vitrified solutions,^31,34^ as well as Raman experiments on hyperquenched solutions.^33^ It entails that dilute LiCl solutions always separate into a pure water and a CS domain upon cooling. When cooling at ambient pressure, the pure water domain is LDA and when cooling at high pressure, it is HDA. Hyperquenched solutions would form a heterogeneous glass, which would persist even after high-pressure treatment. For HDA-type samples of all compositions, we would need to observe a glass transition *T*_g,2_ ≈ 115 K, stemming from pure water HDA accompanied by another (more pronounced) one pertaining to CS embedded in the HDA matrix at *T*_g,CS_^in HDA^ ≈ 140 K (see Figure 11A) upon heating. The scenario A described in Figure 11A is only partly consistent with our observations. Indeed, *T*_g,2_ always remains near 115 K, and HDA could, thus, be devoid of LiCl. Yet, the glass transition of CS in the HDA matrix *T*_g,CS_^in HDA^ cannot be found in our calorimetry scans: the sample pre-emptively transforms to the low-density state. For these LDA-type samples, we would expect two glass transitions: one from pure water LDA at ∼136 K and one from CS embedded in the LDA matrix *T*_g,CS_^in LDA^ at ∼140 K (see Figure 11A) in scenario A. However, we do not see any evidence for two glass transitions in our traces (blue lines in Figure 7) up to the cold crystallization at >150 K. Instead, we only observe the feeble *T*_g,1_ from LDA, which does not remain at 136 K but even shifts to ∼128 K at 4.3 mol % LiCl (R = 22.3). Moreover, we find no signs of such a phase separation in the hyperquenched solutions upon examining our diffraction data (see section 3.2). This is especially striking since a Raman study on hyperquenched solutions demonstrates the coexistence of two states via linear combination.^33^ We believe this apparent discrepancy could possibly have two reasons: (i) Raman spectroscopy is particularly sensitive to the local order, where typically only one or two bond lengths are probed. By contrast, XRD rather probes long-range order extending to 10 bond lengths or more. The mixture could then have phase-separated on a nanoscale level not resolved by conventional diffraction methods, where the shift in *T*_g,1_ is a consequence of longer-range electrostatic forces exerted by nanosegregated ions. (ii) Another possibility is that we implemented an optimized hyperquenching setup^38^ compared to the originally published one,^61^ which allows for cooling rates exceeding 10^6^ K s^–1^. This is important because perhaps already slightly lower cooling rates could lead to partial crystallization of the solution. Crystallization is known to result in a phase-separated solution (see, e.g., ref (19)) consisting of ice and CS. Yet, small contaminations of ice I might be difficult to distinguish from LDA in Raman spectroscopy due to their structural similarity. That is, a small fraction of solution might have crystallized during cooling in the quenching experiment by Suzuki, resulting in a mixture of LDA, CS, and ice I. Ice I is very hard to discriminate from bulk LDA by means of Raman linear combination, because their local coordination geometry is essentially the same. That is, we exclude scenario A for our experiments, but not for other vitrification experiments using cooling rates <10^6^ K s^–1^.

The second scenario shown in Figure 11B suggests that solvent water around LiCl ions always occupies a disordered, high-density state due to electrostrictive forces. Consequently, LiCl can only be incorporated in HDA but not in LDA. This view is consistent with dilatometry and Raman experiments of refs (23, 32, 48) on pressure-vitrified solutions and Raman experiments of ref (33) on hyperquenched solutions. This would imply phase separation into LDA and LiCl-CS even upon hyperquenching. Upon pressurization, the two domains would then reunite again to produce homogeneous LiCl-HDA. When reheating at ambient pressure, homogeneous HDA-LiCl should split via a polyamorphic phase separation into a heterogeneous glass consisting of pure LDA and CS. Similarly to the scenario discussed before, we would then expect to see indications of phase separation of the LDA-type samples in our calorimetry traces or diffraction data. However, we do not see any evidence for this. In contrast, we cannot think of a natural explanation regarding how our observation of only one single *T*_g_ at ∼128 K after the polyamorphic transition fits to this scenario. For a heterogeneous glass composed of two individual components, we would expect a feeble glass transition *T*_g,1_ at 136 K (LDA) and a massive glass transition *T*_g,CS_^in LDA^ at 140 K (CS embedded in LDA, see Figure 11B), in disagreement with our measurements. That is, we also rule out scenario B to explain our observations.

According to the third scenario displayed in Figure 11C, LiCl can be integrated in both LDA and HDA up until the polyamorphic signatures vanish at ∼5.8 mol % (*R* = 16.2). Above ∼5.8 mol %, a homogeneous CS forms where there is a difference of ∼12 K between *T*_g,CS_ and *T*_g,1_. This perception is in agreement with the calorimetry experiments on hyperquenched solutions of ref (17). In this scenario, we expect homogeneously vitrified LiCl-LDA upon hyperquenching the solutions, which then transform into HDA-LiCl once pressurized. Upon reheating, we expect to detect only *T*_g,2_ for HDA-type samples and only *T*_g,1_ for LDA-type samples. All of this is exactly what we observe. While, in principle, both *T*_g_ values could be influenced by the presence of LiCl, only *T*_g,1_ is shifted to lower values and *T*_g,2_ remains hardly affected. We believe this is because the ions can more easily perturb the rather ordered tetrahedral network of LDA. Also when considering the old idea that the addition of salt has the same effects as applying pressure,^62^ it seems reasonable that LDA representing the low-pressure polyamorph is more affected by LiCl than HDA representing the high-pressure polyamorph. This is further manifested in XRD data where the halo peak maximum of LDA is shifted toward higher angles with increasing mole fraction, which speaks in favor of denser local structures. As a result, we infer that only scenario C concurs with all our experimental observations made for pressurized hyperquenched LiCl solutions.

Interestingly, scenario B is consistent with results obtained from pressure-vitrified solutions. The underlying reasons are difficult to deduce, but it seems most likely that also in this experiment cooling rates were too low to reach the homogeneously vitrified state. Furthermore, the individual studies not only use completely different sample preparation pathways (hyperquenching versus pressure-vitrification) but also different, complementary methods for structural analysis (X-ray diffraction vs Raman spectroscopy). In order to resolve these discrepancies, we suggest more extensive studies where samples made from hyperquenching are investigated using spectroscopy and samples made from pressure-vitrification procedures are probed using diffraction.

In summary, we here present a study in which homogeneous vitrification of dilute LiCl solutions is achieved, reaching the LiCl-LDA state. The vitrified glass remains homogeneous even after experiencing the polyamorphic transition at 77 K upon pressurization beyond 1 GPa, turning it into LiCl-HDA. Homogeneous LiCl-HDA then transforms back to homogeneous LiCl-LDA upon reheating at ambient pressure, reaching a state indistinguishable from the state directly after hyperquenching (without any pressurization). This path independence is intriguing and typical of phase transformations ending in an equilibrated phase. For glasses, the associated equilibrated phase is the supercooled liquid. This suggests that, upon heating LiCl-HDA at ambient pressure, it first turns into the deeply supercooled liquid above 115 K. LiCl-HDL then experiences the polyamorphic transition at 139–141 K, which ends in the equilibrated LiCl-LDL phase. This is consistent with both glass transition temperatures being well below 140 K and calorimetric relaxation times of <5 s for both LiCl-LDL and LiCl-HDL, as extracted from our rate-dependent calorimetry study.

## Supporting Information Available

Dilatometric curves without correction for compressibility; the thermograms, as well as Δ*c*_p_ and Δ*T*/*T*_g_ for the other heating rates; and, finally, the glass activation energies that were determined from the calorimetric data (PDF)
