# Supplementary material for: Glass Polymorphism in Hyperquenched Aqueous LiCl Solutions
Source: J Phys Chem B. 2023 Apr 7;127(15):3463–77. doi: 10.1021/acs.jpcb.3c01030 (PMC10123659; doi:10.1021/acs.jpcb.3c01030)
Supplement: Supplementary file 1 — jp3c01030_si_001.pdf [file jp3c01030_si_001.pdf]

Glass Polymorphism in Hyperquenched Aqueous LiCl Solutions

Authors: Johannes Giebelmann, Johannes Bachler, Thomas Loerting*

Affiliations:
Institute of Physical Chemistry, University of Innsbruck, Innrain 52c, A-6020 Innsbruck, Austria

* Author to whom correspondence should be addressed: thomas.loerting@uibk.ac.at

Thomas Loerting – https://orcid.org/0000-0001-6694-3843
Johannes Bachler – https://orcid.org/0000-0002-7071-4952
Johannes Giebelmann - https://orcid.org/0000-0003-4529-5124

Supplementary Material

This supplementary material contains 6 additional figures and one table showing raw data from the dilatometry experiments, additional X-ray diffraction data and additional calorimetric data. The data depicted in each figure (or table) is described and interpreted in the corresponding caption.

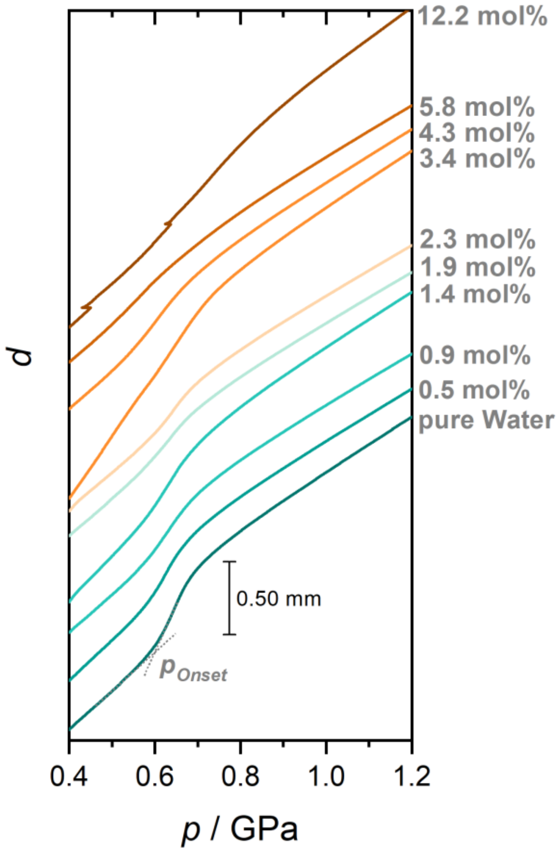

Figure S1: Piston displacement *d* as a function of the pressure *p* during the isothermal compression of HGW, hyperquenched LiCl-solutions (0.5-5.8 mol%) and a CS (12.2 mol%) at 77 K. The curves are offset for clarity and not normalized since the sample mass cannot be determined. In contrast to the data shown in figure 1 in the main text, no straight line was subtracted to account for the compressibility at ~1 GPa. This means that the step-like densification with the onset pressure p_{Onset} appears less pronounced.

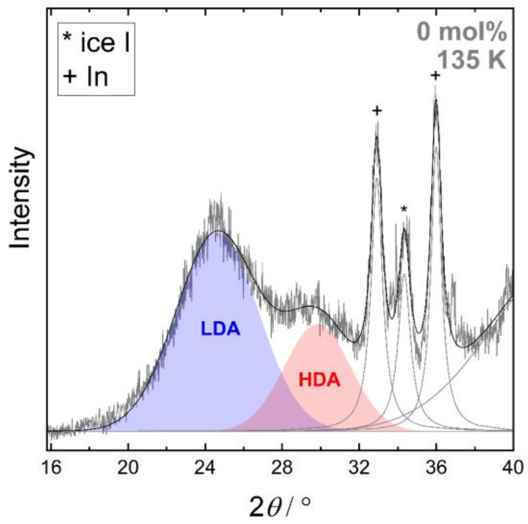

Figure S2: Deconvolution of the diffractogram of pure water HDA made from HGW recorded at 135 K (also shown in figure 3). Halo peaks were described using gaussian functions while bragg-reflexes were fitted with Lorentz curves. Based on the peak position, two halo peaks (one for LDA and one for HDA) are identified and labeled accordingly.

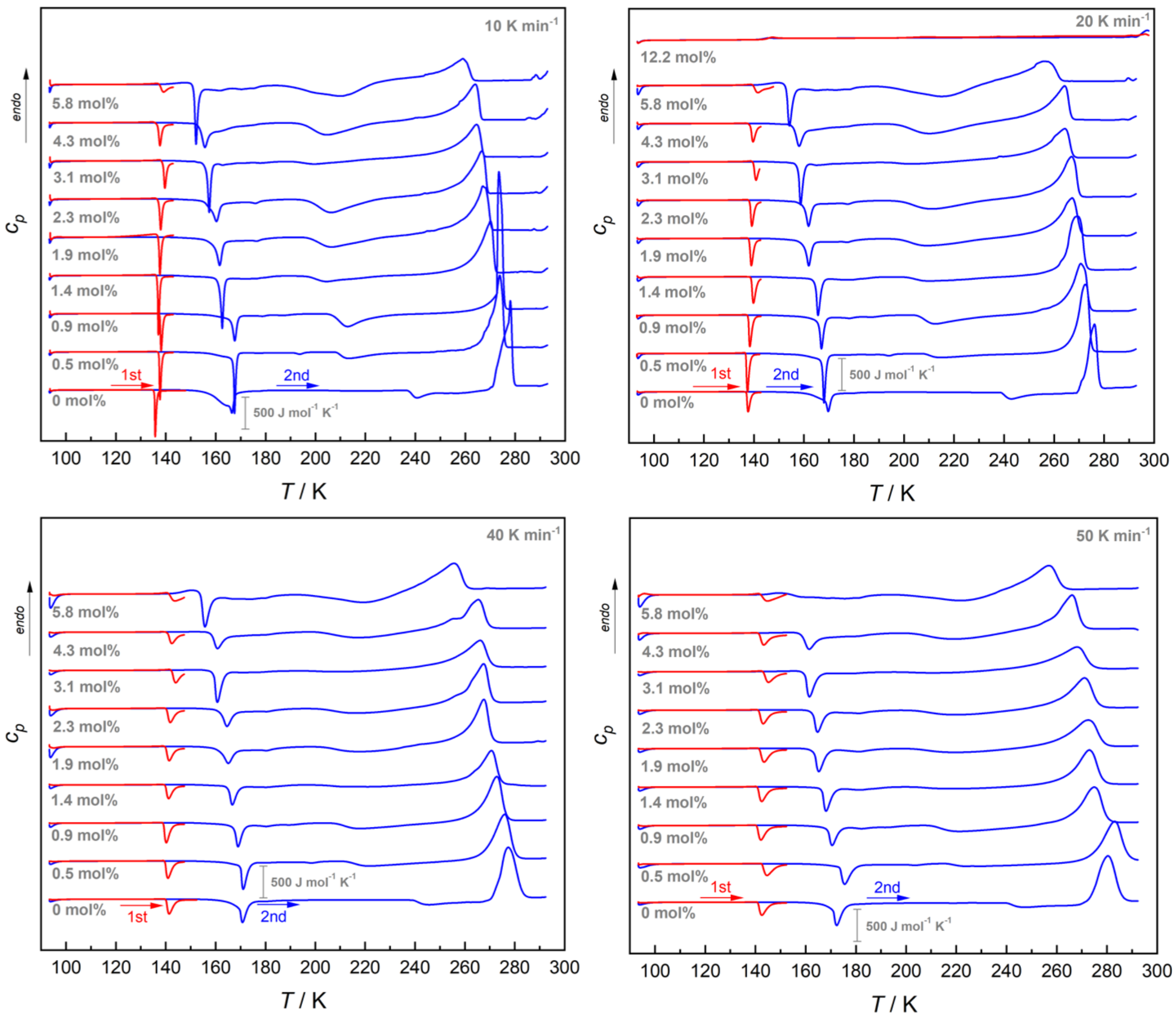

Figure S3: First (red) and second (blue) DSC heating traces of LiCl-HDA made from hyperquenched LiCl-solutions and a reference CS (12.2 mol%) for the heating rates 10, 20, 40 and 50 K min^{-1}. Curves are offset for clarity and are normalized to the amount of water using the melting endotherm as described in the methods section. In the text, the polyamorphic behavior at 30 K min^{-1} is discussed thoroughly. The same behavior is reproduced at the other rates: At around 140 K, LiCl-HDA transforms to LiCl-LDA which causes the exothermic feature in the red traces. Upon re-cooling and heating LiCl-LDA, the cold-crystallization to ice I_{sd} occurs as can be deduced from the exotherm in the blue traces between 160 and 180 K. The

exotherm at ~140 K upon intially heating the densified 5.8 mol% samples stems from enthalpy relaxation rather than a polyamorphic transition, as discussed in the main text. However, due to the high water content, cold-crystallization is still observed as a pronounced exotherm at ~160 K. In the case of the 5.8 mol% sample at 50 K min^{-1}, this sharp exotherm is missing. Instead, a series of very broad peaks is noted. From this we infer that the sample already partially crystallized after the first heating scan. The CS (12.2 mol%) shows no polyamorphic transition and no cold-crystallization.

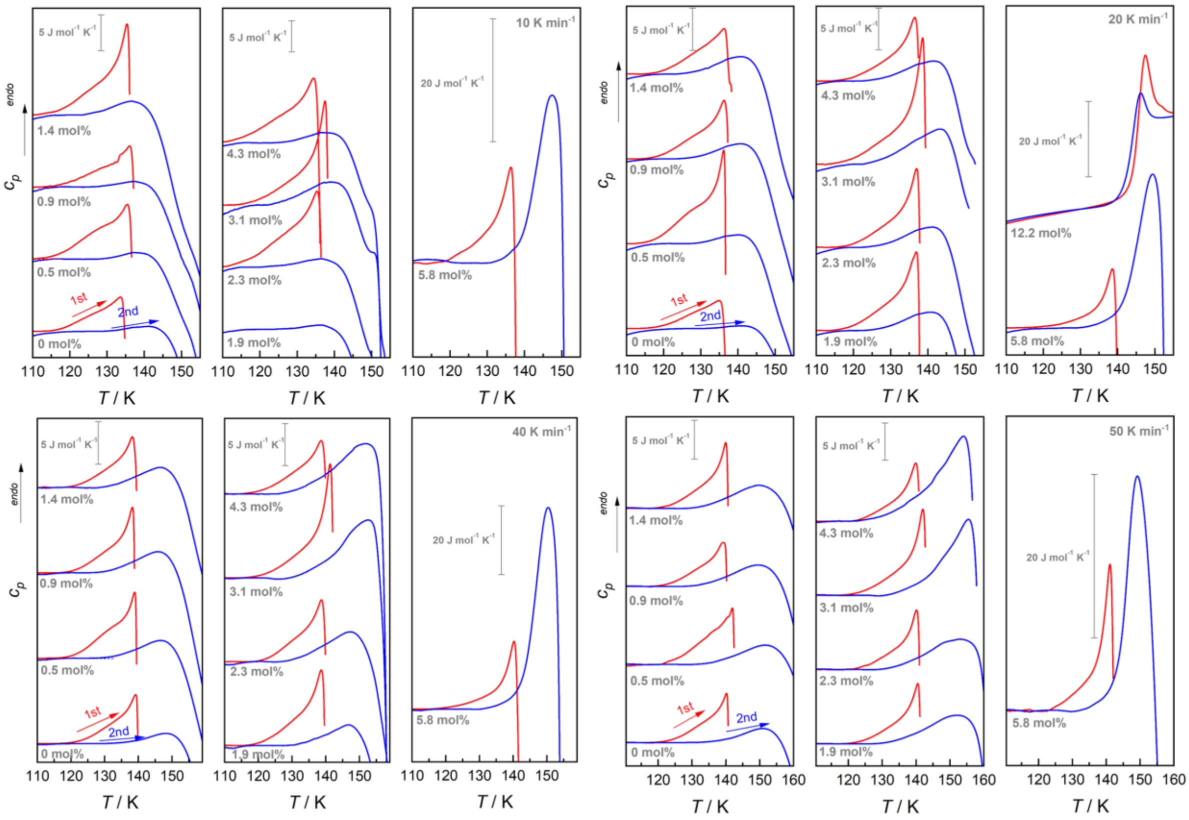

Figure S4: Magnification of figure S3 with a focus on the glass transitions of LiCl-HDA (red) and LiCl-LDA (blue). The overall appearance of the glass transitions at these rates is very similar to the glass transitions observed at 30 K min^{-1} which is discussed thoroughly in the results and discussion section. Increasing the heating rate leads to an increase in the glass transition temperature which has already been shown in the main text. Additionally, the spike-like feature is observed at every rate for LiCl-HDA between 0 and 4.3 mol%.

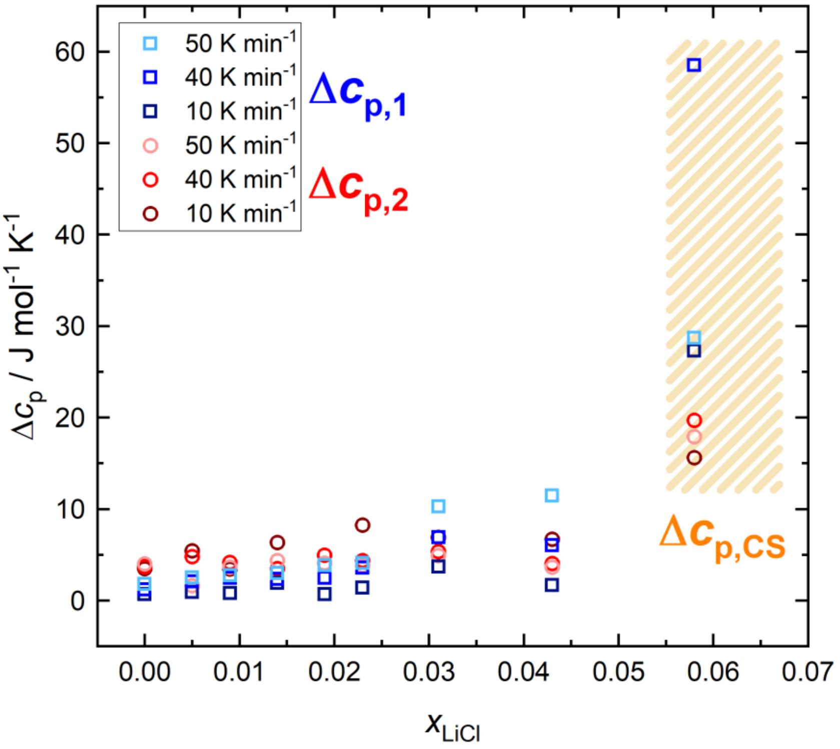

Figure S5: Changes in heat capacity Δc_p at the glass transition of LiCl-HDA ($\Delta c_{p,2}$, colored red), LiCl-LDA ($\Delta c_{p,1}$, colored blue), and LiCl-CS ($\Delta c_{p,CS}$, colored orange) for heating rates of 10, 40 and 50 K min^{-1} as a function of the concentration. The values were determined from the curves shown in figure S3 as described in the main text. Error bars are omitted for clarity. The error estimated for Δc_p is roughly 0.4 J mol^{-1} K^{-1}. As observed for the rates shown in the paper, $\Delta c_{p,2}$ only shows little concentration dependency while $\Delta c_{p,1}$ increases slightly towards higher mole fractions. Additionally, there is a pronounced jump between 4.3 mol% and 5.8 mol% that marks the switch to a solute-governed behavior.

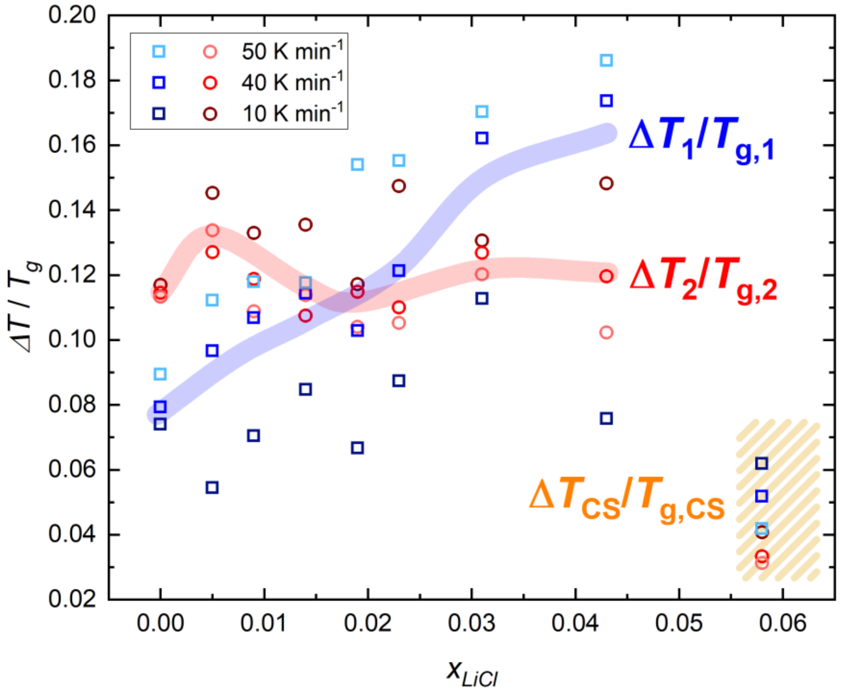

Figure S6: Relative width $\Delta T/T_g$ of the glass transition of LiCl-HDA ($\Delta T_2/T_{g,2}$, colored red), LiCl-LDA ($\Delta T_1/T_{g,1}$,, colored blue), and LiCl-CS ($\Delta T_{CS}/T_{g,CS}$, colored orange) as a function of the concentration at heating rates of 10, 40 and 50 K min^{-1}. Thick transparent lines serve as guides to the eye. The values were determined as described in the main text. The error estimated for $\Delta T/T_g$ is ~0.01. Error bars were omitted for clarity. Although the values scatter quite strongly, a similar behavior as found at 20 and 30 K min^{-1} in the main text is observed here. That is, $\Delta T_2/T_{g,2}$ shows no clear trend while $\Delta T_1/T_{g,1}$ increases when increasing the mole fraction of LiCl. The width of the glass transition of the CS is rather small compared to the one of LiCl-HDA and -LDA.

Table S1: Glass activation energies for the glass transition of LiCl-HDA $E_{g,2}$, LiCl-LDA $E_{g,1}$, the densified CS $E_{g,\text{densified CS}}$, and the at ambient pressure annealed CS $E_{g,CS}$ along with the estimated error. The corresponding relaxation times τ_g at the respective glass transition temperatures are shown as well. The energies were determined from the heating rate dependency of $T_{g,2}$ and $T_{g,1}$ respectively. Therefore, the natural logarithm of the heating rate *ln(q)* was plotted as a function of the inverse glass transition temperature $1/T_g$. The data was fitted using a linear function which is a linearized form of Equation 1 in the main text. From the slope $-E_g/R$, the values for E_g were calculated. The error was estimated based on the error of the slope obtained from the fit using gaussian error propagation. The relaxation times were calculated using Equation 2 in the main text.

x_{LiCl}	$E_{g,2}$ / kJ	$E_{g,1}$ / kJ	*Error* $E_{g,2}$ / kJ	*Error* $E_{g,1}$ / kJ	$\tau_{g,2}$/ s	$\tau_{g,1}$ / s	*Error* $\tau_{g,2}$ / s	*Error* $\tau_{g,1}$ / s
0	27.3	33.7	2.8	1.7	8.8	9.1	0.9	0.5
0.005	23.5	29.5	3.9	9.0	10.4	10.0	1.7	3.1
0.009	32.4	39.0	5.5	7.9	7.4	7.3	1.3	1.5
0.014	29.3	29.1	1.8	3.4	8.3	9.7	0.5	1.1
0.019	26.8	31.8	5.7	12.7	9.0	8.8	1.9	3.5
0.023	22.4	29.5	1.5	5.2	10.9	9.3	0.7	1.6
0.031	25.4	27.0	9.8	5.9	9.5	10.2	3.7	2.2
0.043	19.9	64.6	3.1	13.4	12.4	4.3	1.9	0.9
x_{LiCl}	$E_{g,\text{densified CS}}$ / kJ	$E_{g,CS}$ / kJ	*Error* $E_{g,\text{densified CS}}$ / kJ	*Error* $E_{g,CS}$ / kJ	$\tau_{g,\text{densified CS}}$ / s	$\tau_{g,CS}$ / s	*Error* $\tau_{g,\text{densified CS}}$ / s	*Error* $\tau_{g,CS}$ / s
0.058	89.8	58.4	2.1	4.2	3.4	5.8	0.1	0.4
